# Supplementary material for: Do components of adult height predict body composition and cardiometabolic risk in a young adult South Asian Indian population? Findings from a hospital-based cohort study in Pune, India: Pune Children’s Study
Source: BMJ Open. 2020 Oct 7;10(10):e036897. doi: 10.1136/bmjopen-2020-036897 (PMC7542941; doi:10.1136/bmjopen-2020-036897)

Supplementary Figure 1: Matrix showing correlations between height components and regional body composition measures

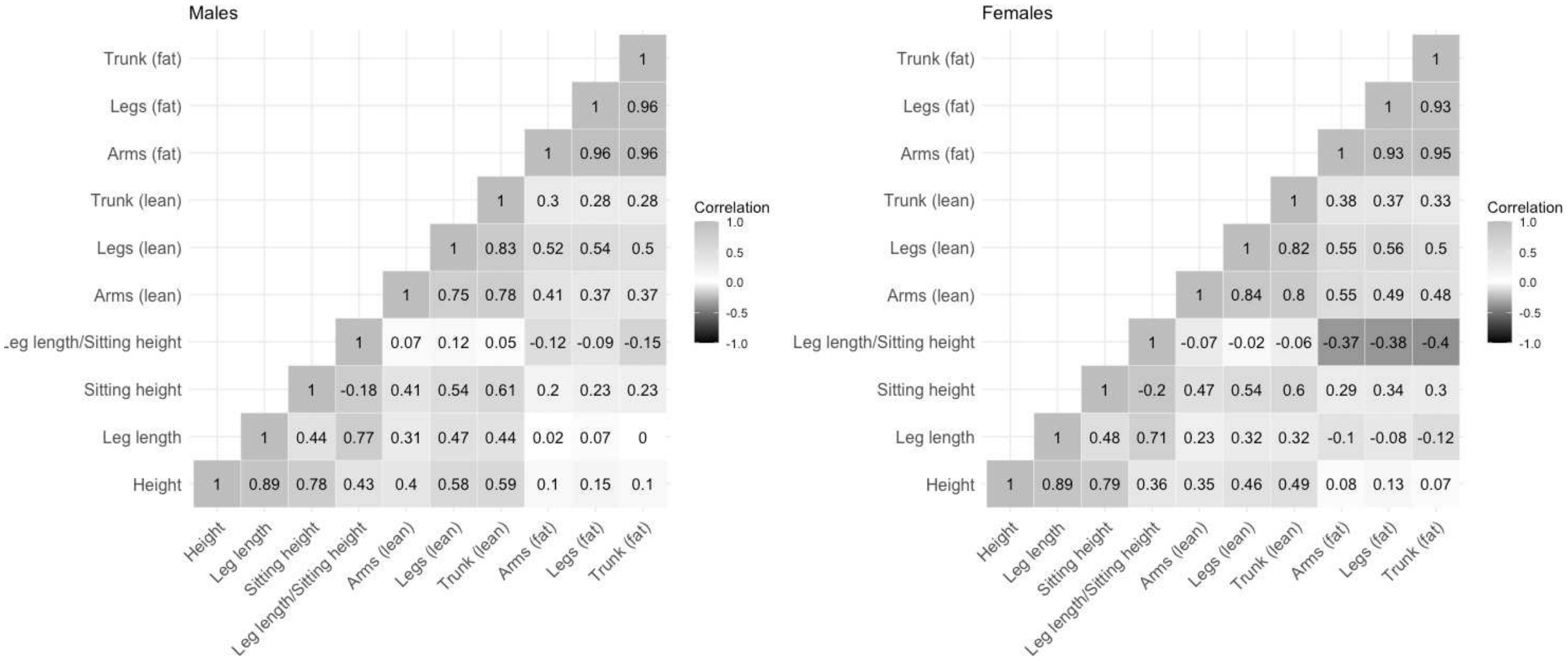

Supplementary Figure 2: Relationships of leg length and sitting height with selected CVD risk factors

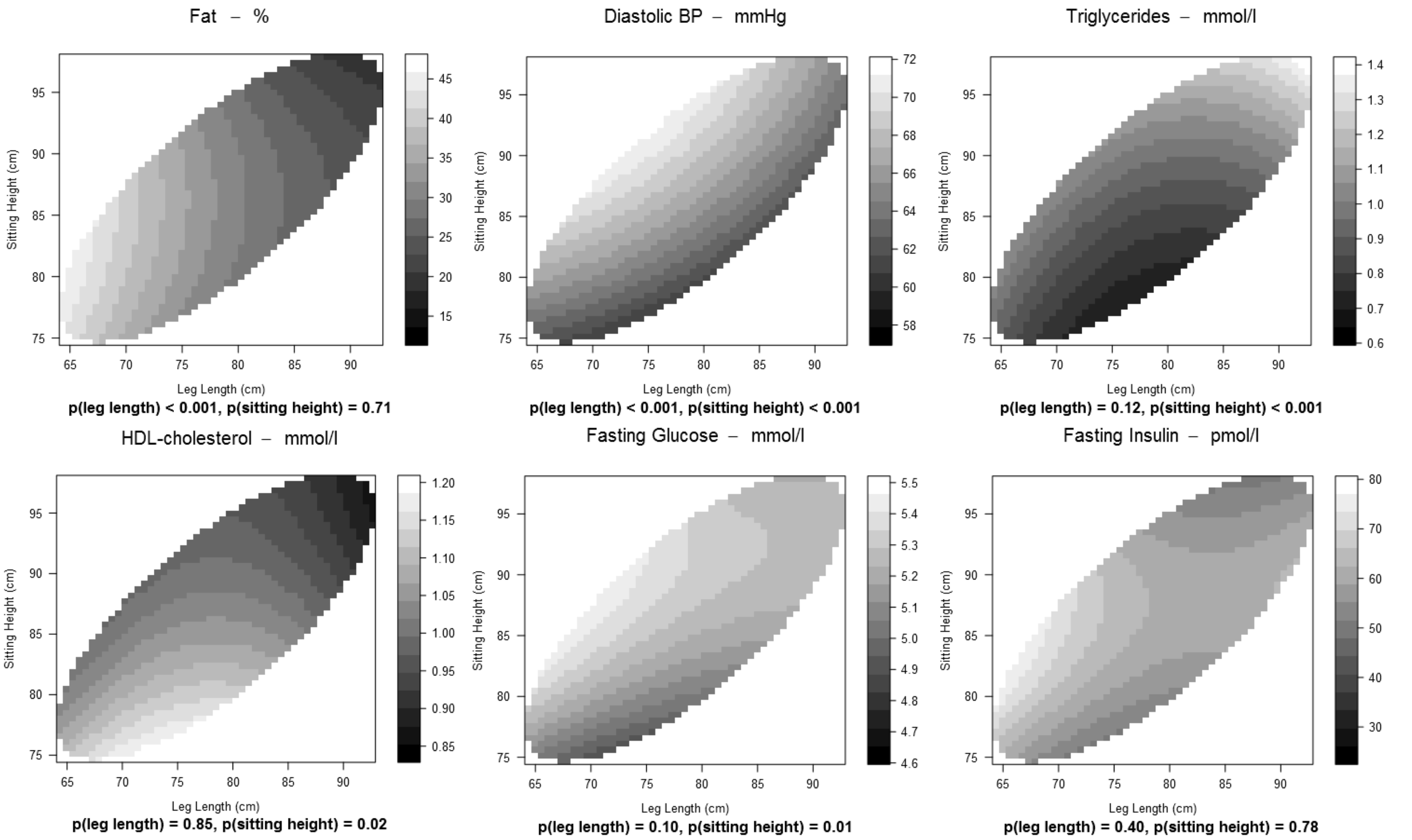

Supplementary Figure 3: Relationships of leg length and sitting height with selected CVD risk factors

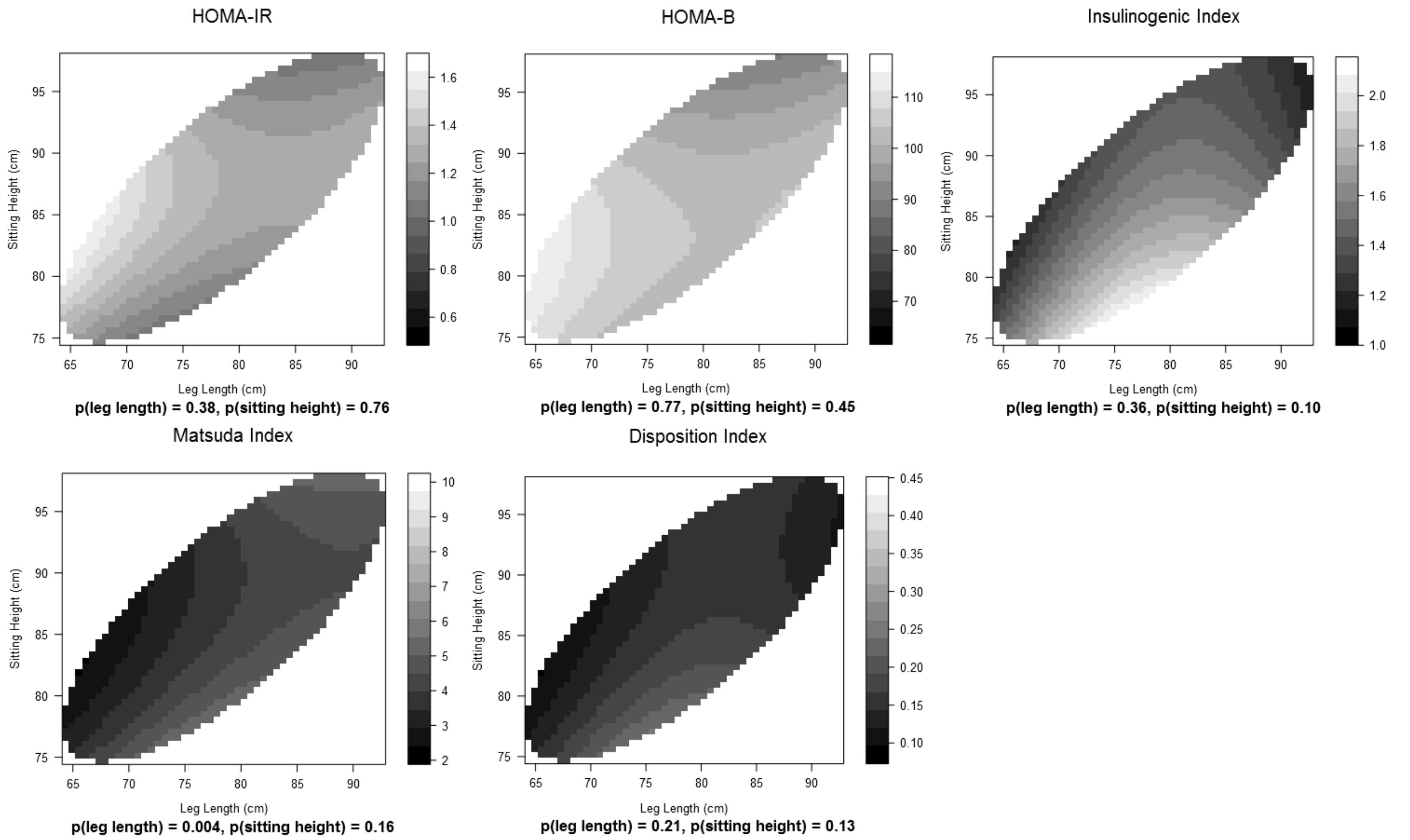

Supplement: Supplementary data [file bmjopen-2020-036897supp001.pdf]
