# Supplementary material for: Do components of adult height predict body composition and cardiometabolic risk in a young adult South Asian Indian population? Findings from a hospital-based cohort study in Pune, India: Pune Children’s Study
Source: BMJ Open. 2020 Oct 7;10(10):e036897. doi: 10.1136/bmjopen-2020-036897 (PMC7542941; doi:10.1136/bmjopen-2020-036897)
Supplement: Author's manuscript [file bmjopen-2020-036897.draft_revisions.pdf]

BMJ Open is committed to open peer review. As part of this commitment we make the peer review history of every article we publish publicly available.

When an article is published we post the peer reviewers' comments and the authors' responses online. We also post the versions of the paper that were used during peer review. These are the versions that the peer review comments apply to.

The versions of the paper that follow are the versions that were submitted during the peer review process. They are not the versions of record or the final published versions. They should not be cited or distributed as the published version of this manuscript.

BMJ Open is an open access journal and the full, final, typeset and author-corrected version of record of the manuscript is available on our site with no access controls, subscription charges or pay-per-view fees (http://bmjopen.bmj.com).

If you have any questions on BMJ Open's open peer review process please email info.bmjopen@bmj.com

BMJ Open

COMPONENTS OF ADULT HEIGHT AS PREDICTORS OF BODY COMPOSITION AND CARDIOMETABOLIC RISK IN A YOUNG ADULT INDIAN POPULATION; THE PUNE CHILDREN'S STUDY

Journal:	*BMJ Open*
Manuscript ID	bmjopen-2020-036897
Article Type:	Original research
Date Submitted by the Author:	18-Mar-2020

Complete List of Authors:	Kumaran, Kalyanaraman; University of Southampton, MRC Lifecourse Epidemiology Unit; CSI Holdsworth Memorial Hospital, Epidemiology Research Unit<br>Joshi, Suyog; KEM Hospital Pune, Diabetes Unit<br>Di Gravio, Chiara; Vanderbilt University<br>Lubree, Himangi; KEM Hospital Pune, Vadu Rural Health Centre<br>Joglekar, Charudatta ; BKL Walawalkar Rural Medical College, Regional Centre for Adolescent Health and Nutrition<br>Bhat, Dattatray; KEM Hospital Pune, Diabetes Unit<br>Kinare, Arun; Bharati Vidyapeeth Deemed University Medical College, Department of Radiology<br>Bavdekar, Ashish; KEM Hospital Pune, Department of Paediatrics<br>Bhave, Sheila; KEM Hospital Pune, Department of Paediatrics<br>Pandit, Anand; KEM Hospital Pune, Department of Paediatrics<br>Osmond, Clive; University of Southampton,<br>Yajnik, Chittaranjan; KEM Hospital Pune, Diabetes Unit<br>Fall, Caroline ; University of Southampton, MRC Lifecourse Epidemiology Unit

Keywords:	Cardiac Epidemiology < CARDIOLOGY, EPIDEMIOLOGY, DIABETES & ENDOCRINOLOGY, PREVENTIVE MEDICINE

SCHOLARONE™ Manuscripts

I, the Submitting Author has the right to grant and does grant on behalf of all authors of the Work (as defined in the below author licence), an exclusive licence and/or a non-exclusive licence for contributions from authors who are: i) UK Crown employees; ii) where BMJ has agreed a CC-BY licence shall apply, and/or iii) in accordance with the terms applicable for US Federal Government officers or employees acting as part of their official duties; on a worldwide, perpetual, irrevocable, royalty-free basis to BMJ Publishing Group Ltd ("BMJ") its licensees and where the relevant Journal is co-owned by BMJ to the co-owners of the Journal, to publish the Work in this journal and any other BMJ products and to exploit all rights, as set out in our licence.

The Submitting Author accepts and understands that any supply made under these terms is made by BMJ to the Submitting Author unless you are acting as an employee on behalf of your employer or a postgraduate student of an affiliated institution which is paying any applicable article publishing charge ("APC") for Open Access articles. Where the Submitting Author wishes to make the Work available on an Open Access basis (and intends to pay the relevant APC), the terms of reuse of such Open Access shall be governed by a Creative Commons licence – details of these licences and which Creative Commons licence will apply to this Work are set out in our licence referred to above.

Other than as permitted in any relevant BMJ Author's Self Archiving Policies, I confirm this Work has not been accepted for publication elsewhere, is not being considered for publication elsewhere and does not duplicate material already published. I confirm all authors consent to publication of this Work and authorise the granting of this licence.

For peer review only

COMPONENTS OF ADULT HEIGHT AS PREDICTORS OF BODY COMPOSITION AND CARDIOMETABOLIC RISK IN A YOUNG ADULT INDIAN POPULATION; THE PUNE CHILDREN'S STUDY

Kumaran K[1,2], Joshi SM[3], Di Gravio C[1], Lubree H[4], Joglekar C[5], Bhat DS[3], Kinare AS[3], Bavdekar A[4], Bhave SA[6], Pandit AN[6], Osmond C[1], Yajnik CS[3], Fall CHD[1]

[1]MRC Lifecourse Epidemiology Unit, University of Southampton, Southampton, UK.

[2]Epidemiology Research Unit, CSI Holdsworth Memorial Hospital, Mysore, India

[3]Diabetes Unit, KEM Hospital Research Centre, Pune, India.

[4]Vadu Rural Health Program, KEM Hospital Research Centre, Pune, India

[5]Department of Statistics, BKL Walawalkar Hospital and Diagnostic Centre, Ratnagiri, India

[6]Department of Pediatrics, KEM Hospital, Pune, India.

Corresponding author: Chittaranjan S. Yajnik, Kamalnayan Bajaj Diabetology Research Centre, Diabetes Unit, King Edward Memorial Hospital Research Centre, Rasta Peth, Pune 411011, India

Telephone: +91-20-26061958
Fax: +91-20-26111958
Email: diabetes@kemdiabetes.org; csyajnik@gmail.com

WORD COUNT ABSTRACT: 233

WORD COUNT MAIN TEXT: 2989

For peer review only

Abstract

Objectives: We investigated whether the relationship between components of height and CVD risk may be explained by body composition. We also examined relationships between parental heights and offspring CVD risk.

Design: A prospective cohort study.

Setting: A secondary care hospital setting in Pune, India

Participants: We studied 357 young adults and their parents in the Pune Children's Study.

Primary and secondary outcomes: We measured weight, total height, leg length, sitting height, plasma glucose, insulin and lipids, and blood pressure. Total and regional lean and fat mass were measured by dual x-ray absorptiometry.

Results: Leg length was inversely, and sitting height directly related to BMI. Total height and leg length were directly related to lean mass while sitting height was directly related to both lean and fat mass. Leg length was inversely related to systolic blood pressure and 120-minute glucose, independent of lean and fat mass. Sitting height was directly related to systolic blood pressure and triglycerides; these relationships were attenuated on adjustment for lean and fat mass. When examined simultaneously, greater leg length was protective and greater sitting height was associated with a more detrimental CVD risk profile.

Conclusions: Shorter adult leg length and greater sitting height are associated with a more adverse CVD risk factor profile. The mechanisms need further study but our findings suggest a role for lean and fat mass.

Key words: adult height; leg length; sitting height; intergenerational change; cardiometabolic risk factors; India

For peer review only

Strength and limitations of the study:

- One of few birth cohorts in a low- and middle-income country setting with data on parental and offspring height, and height components
- Data available on body composition measurements using DXA
- Glucose-insulin measurements available as part of OGTT
- Participants born in one hospital in Pune, India
- ~25% loss to follow-up between childhood and young adulthood.

What is already known on this subject:

Longer leg length has been associated with lower cardiovascular disease risk factors while greater sitting height has either shown no association or an association with greater cardiovascular risk.

Much of the evidence is from high-income countries while evidence from low- and middle-income country settings is limited.

Reasons for the association between height and cardiovascular risk remain unknown.

What this study adds:

This study examined whether the association between height components and cardiovascular risk could be explained by body composition, using direct measurements of fat and lean mass.

The associations with leg length and sitting height may reflect the protective benefits of better growth in early childhood and the detrimental effects of accelerated growth in later childhood respectively.

These relationships are partially explained by body composition.

For peer review only - http://bmjopen.bmj.com/site/about/guidelines.xhtml

Introduction

Short adult height is associated with an increased risk of cardiovascular disease (CVD) and its risk factors in both high-income and low- and middle-income countries (LMICs),[1-4] although the association is less consistent in the latter[5,6].The reasons for the association remain unknown. Intrauterine and infant growth are determinants of final height[7,8]. Impaired fetal and infant growth are also associated with CVD[9]. It is therefore possible that the association between height and CVD is related to early life programming. Short height may also reflect lower socio-economic class,[10] and deprivation throughout childhood. The relationship between height and CVD may have a genetic basis; height is partly genetically determined as are some CVD risk factors.

Longer leg length has been associated with lower CVD risk,[11-14], while greater sitting height has shown either no association,[11,12] or an association with higher risk[14]. Shorter leg length and greater sitting height have also been associated with greater body mass index (BMI) and/or higher body fat percent[15,16].

The Pune Children's Study (PCS) was set up to prospectively study early life antecedents of adult disease[17]. At 8 years of age, taller children had a more adverse CVD risk profile[19]. We have now investigated the relationship of final height and its components with CVD risk factors at 21 years of age (height components were not assessed at 8 years). We speculated that body composition may explain the relationship between height components and CVD risk. To our knowledge, this has not been examined before. We also investigated CVD risk factors in relation to parental height and intergenerational change in height.

Methods

For peer review only - http://bmjopen.bmj.com/site/about/guidelines.xhtml

The PCS[17] is an urban cohort of 477 full-term singleton babies who were born in the KEM Hospital, Pune during 1987-1989. During 2009-11, the cohort members were invited for further studies. They were admitted to our unit the evening before the investigations and fasted overnight after a standard dinner.

Anthropometry and body composition: Weight was measured to the nearest 5g using an electronic scale (Conweigh Electronic Instruments, Mumbai). Standing and sitting height were measured to the nearest 0.1 cm using a wall-mounted stadiometer. Leg length was computed by subtracting sitting height from standing height. The predicted height of the offspring (mid-parental height) was derived using a standard formula (boys: [{maternal height+paternal height}/2] + 7 cm; girls: [{maternal height+paternal height}/2] - 7 cm[18]. The intergenerational change in height between children and parents was calculated by subtracting mid-parental height from offspring adult height. Participants' total and regional body fat and lean mass were assessed using dual energy X-ray absorptiometry (DXA; Lunar Prodigy, GE, USA).

CVD risk factors: Blood pressure was measured using a digital monitor (UA 767PC, A & D Instruments Ltd, UK); the average of two readings made five minutes apart was used. Plasma lipids, glucose and insulin were measured on fasting venous blood. An oral glucose tolerance test was carried out according to the WHO protocol,[19] followed by further blood samples at 30 and 120 minutes for glucose and insulin.

The Standard of Living Index (SLI)[20] was used to assess socio-economic status. It is a standardized questionnaire based on information about housing, amenities and possessions; higher scores indicate higher social class. Ethical permission was obtained from the KEM Hospital Ethics Committee and informed consent was obtained from all participants.

For peer review only - http://bmjopen.bmj.com/site/about/guidelines.xhtml

Laboratory analyses*:* Plasma glucose, cholesterol, HDL-cholesterol, and triglyceride concentrations were measured using enzymatic methods (Hitachi 902, Germany). Between-batch coefficients of variation for all these assays were <3% in the normal range. Plasma insulin was measured using a Delfia technique (Victor 2, Wallac, Finland); between-batch coefficients of variation were <6%. Insulin resistance (HOMA-IR) and beta-cell function (HOMA-B) were calculated using the online Oxford model[21]. Insulin secretion was measured as Insulinogenic index (increment in plasma insulin/increment in plasma glucose at 30 min)(22]. The Matsuda index of insulin sensitivity was computed by k/sqrt(fasting glucose*120 min glucose*fasting insulin*120 min insulin) where k=10,000[23]. Disposition index was calculated using HOMA-B and HOMA-IR, to reflect beta-cell function for concurrent insulin resistance[24].

Definitions: Overweight was defined as BMI ≥25 kg/m^2 and <30 kg/m^2, and obesity as BMI ≥30 kg/m^2[25]. Stunting was defined as a height Z-score of less than -2 below the CDC average[26]. Hyperglycaemia was defined as either impaired fasting glucose (IFG, fasting plasma glucose 5.6-6.9 mmol/L) or impaired glucose tolerance (IGT, 120-minute plasma glucose 7.8-11.0 mmol/L) or diabetes mellitus (DM, fasting plasma glucose ≥7.0 mmol/L or 120-minute plasma glucose ≥11.1 mmol/L)[27]. Hypercholesterolaemia was defined as plasma total cholesterol ≥5.1 mmol/L, [28] hypertriglyceridaemia as plasma triglyceride concentration ≥1.7 mmol/L and low HDL-cholesterol as HDL-cholesterol concentration <1.03 mmol/L for men and <1.29 mmol/L for women[29]. Hypertension was defined as systolic blood pressure ≥130 mmHg or diastolic blood pressure ≥85 mmHg[29].

Statistical Methods: Exposure variables (offspring height and its components, parental height, and intergenerational change in height) were converted into Z-scores to enable comparison of

For peer review only - http://bmjopen.bmj.com/site/about/guidelines.xhtml

effects. Associations with body composition were tested using age-and-sex adjusted partial correlations. Multiple linear regression was used to identify associations between height components and CVD risk factors, adjusting for age, sex and age-sex interaction (model 1). We then examined the simultaneous association of height components by further adjusting model 1. To avoid multicollinearity, we fitted two sets of models: i) leg length and sitting height, and ii) total height and leg length to sitting height ratio. Finally, we further adjusted for fat mass, lean mass and their interactions with sex. The estimated regression coefficients (β) represent the change in outcome per SD change in exposure. We derived contour plots for the outcome variables as a function of leg length and sitting height, adjusted for age and sex. We used the Mahalanobis distance to identify the ellipse that contained 95% of the data, and to exclude unlikely combinations of leg length and sitting height.

To test possible selection bias, we compared body size, lipid and glucose-insulin variables between participants and non-participants using regression imputation. We developed an imputation model using multiple regression with variables significantly associated with each other at 8 years. We then applied this model to compare 21-year observed values for participants and imputed values for non-participants.

Statistical analyses were performed using SPSS 21 (SPSS Inc., USA) and R V.3.2.3.

Results

Of 477 children who were studied at 8 years of age, 357 (75%) participated in the 21-year study (191 males). Non-participants had higher 8-year BMI (14.0 v 13.6 kg/m^2, p=0.05), but similar 8-year height, and lipid, glucose and insulin concentrations compared to participants. Participants'

For peer review only - http://bmjopen.bmj.com/site/about/guidelines.xhtml

21-year BMI showed no differences from imputed values for non-participants (21.6 vs 21.9 kg/m^2; p>0.05).

General characteristics:

Both men and women had height CDC Z-scores below the average (Table 1). They were taller than their parents (men by 5.7 cm and women by 4.1 cm) with lower rates of stunting (8.0% and 14.5% of men and women versus 26.8% and 28.5% of fathers and mothers respectively). Compared to their fathers, sons had a greater leg length by 3.7 cm and sitting height by 2.5 cm. Compared to their mothers, daughters had a greater leg length by 2.5 cm and sitting height by 1.3 cm. Offspring height correlated positively with height of both parents (men: r=0.43 and 0.55 for fathers and mothers respectively; women: 0.50 and 0.48; p<0.001 for all). Heights of the parents were positively correlated (r=0.23; p<0.001). At 21 years, 18.5% of participants were overweight, 2.5% were obese, 4.8% were hypertensive, 5.6% had hypercholesterolaemia, 7.6% had hypertriglyceridaemia, and 69.3% had low HDL-cholesterol concentrations. None were on treatment for hypertension or dyslipidemia. Three participants known to have diabetes were on insulin therapy; a further 18.5% were found to be hyperglycaemic (11.2% IFG, 5.9% IGT and 1.4% type-2 DM).

Table 1: Characteristics of the cohort participants

General characteristics	Men (n=191)	Women (n=166)
**Birth data**		
Birth weight (g)	2824 (443)	2721 (481)
**21-year data**		
Age (years)	21.4 (0.4)	21.4 (0.4)
Anthropometry		
Height (cm)	172.0 (6.6)	156.8 (6.4)
CDC Z score	-0.66 (0.92)	-0.99 (0.98)

For peer review only - http://bmjopen.bmj.com/site/about/guidelines.xhtml

Stunted n (%)	15 (8.0)	24 (14.5)
Sitting height (cm)	89.8 (3.2)	82.5 (3.1)
Leg length (cm)	82.2 (4.4)	74.2 (4.3)
Leg length to sitting height ratio	0.91 (0.04)	0.89 (0.04)
Weight (kg)	65.3 (13.1)	52.3 (10.6)
BMI (kg/m^2)	22.0 (4.0)	21.2 (4.1)
Lean mass (kg)	46.5 (5.8)	30.2 (3.9)
Fat mass (kg)	15.7 (9.1)	19.1 (8.0)
Fat percent (%)	22.4 (9.6)	35.2 (8.4)
Cardiometabolic risk factors		
Systolic BP (mmHg)	115.3 (10.1)	103.0 (9.1)
Diastolic BP (mmHg)	65.5 (8.1)	63.7 (7.9)
Cholesterol (mmol/L)	3.83 (0.76)	3.75 (0.74)
Triglycerides (mmol/L)**	0.89 (0.67, 1.25)	0.76 (0.62, 1.03)
HDL cholesterol (mmol/L)	0.97 (0.17)	1.10 (0.22)
Fasting glucose (mmol/L)**	5.16 (4.88, 5.38)	5.00 (4.77, 5.27)
120-minute glucose (mmol/L)**	5.55 (4.83, 6.55)	5.72 (4.88, 6.50)
Fasting insulin (pmol/L)**	41.5 (26.2, 64.2)	48.0(31.9, 68.3)
HOMA-IR	0.8 (0.5, 1.2)	0.9 (0.6, 1.3)
HOMA-B	85.5 (61.1, 106.4)	94.3 (72,7, 122.4)
Insulinogenic Index	1.25 (0.94, 2.10)	1.59 (0.94, 2.43)
Matsuda Index	5.99 (3.81, 8.78)	5.31 (3.55, 8.09)
Disposition Index	0.20 (0.12, 0.33)	0.20 (0.12, 0.36)
**Parental characteristics**		
	Fathers (n=310)	Mothers (n=360)
Age (years)	52.5 (4.2)	46.2 (4.5)
Height (cm)	165.9 (6.1)	153.0 (5.7)
Sitting height (cm)	87.3 (3.3)	81.4 (3.09)
Leg length (cm)	78.6 (4.0)	71.6 (3.7)
CDC Z score	-1.52 (0.84)	-1.58 (0.88)
Stunted n (%)	82 (26.8)	102 (28.5)

Values are mean (SD) unless otherwise stated; ** median (inter-quartile range)

Relationships of height with birth weight, socio-economic status, BMI and body composition:

Total height, leg length and sitting height at 21 years were directly related to birthweight (r=0.39, 0.34 and 0.33 respectively; p<0.001 for all). Higher SLI scores were associated with taller total

height (r=0.11; p<0.05); correlations with leg length and sitting height were similar (0.09 and 0.10; p>0.05). Leg length and leg length to sitting height ratio were inversely related to BMI (r=-0.18; and r=-0.30 respectively) while sitting height was directly related (r=0.17); (p<0.01 for all). Total height and leg length were directly related to lean mass while sitting height was directly related to both lean and fat mass (Table 2). Leg length to sitting height ratio was inversely related to fat mass and body fat percent.

For peer review only

Table 2: Relationship of height and components of height to body composition

	Lean mass (kg)			Fat mass (kg)			Body fat percent		
	β	95% CI	P	β	95% CI	p	β	95% CI	P
**Men**									
Total height (z)	3.6	3.0, 4.3	<0.001	1.2	-0.1, 2.5	0.07	0.1	-1.3, 1.5	0.9
Leg length (z)	2.8	2.1, 3.6	<0.001	0.4	-1.0, 1.7	0.6	-0.5	-1.9, 0.9	0.5
Sitting height (z)	3.6	2.9, 4.3	<0.001	2.1	0.8, 3.4	0.001	1.0	-0.4, 2.4	0.1
Ratio of leg length to sitting height (z)	0.5	-0.3, 1.4	0.2	-1.2	-2.5, 0.1	0.08	-1.4	-2.7, 0.0	0.05
**Women**									
Total height (z)	2.0	1.5, 2.5	<0.001	1.1	-0.1, 2.3	0.08	-0.2	-1.5, 1.1	0.8
Leg length (z)	1.4	0.8, 1.9	<0.001	-0.6	-1.8, 0.7	0.4	-1.6	-2.9, -0.3	0.01
Sitting height (z)	2.3	1.8, 2.8	<0.001	2.8	1.6, 4.0	<0.001	1.5	0.2, 2.9	0.02
Ratio of leg length to sitting height (z)	0.0	-0.6, 0.6	0.9	-2.9	-4.0, -1.7	<0.001	-3.3	-4.5, -2.1	<0.001

Data derived using linear regression and all variables as continuous; All analyses were adjusted for age.

Relationships of height and its components with CVD risk factors:

Relationships between height components and CVD risk factors were similar in both sexes; we therefore present a sex-adjusted combined analysis. Total height was inversely related to 120-minute glucose concentration and Matsuda index (Table 3). Leg length was inversely related to diastolic blood pressure, total cholesterol, fasting and 120-minute glucose concentrations, and

For peer review only - http://bmjopen.bmj.com/site/about/guidelines.xhtml

Matsuda index. Sitting height was directly related to systolic blood pressure and triglycerides. Leg length to sitting height ratio was inversely related to systolic and diastolic blood pressure, total cholesterol, triglycerides, and fasting and 120-minute glucose concentrations, and directly related to Matsuda index. After adjustment for lean and fat mass, the relationships of height and leg length with risk factors remained of similar strength except for Matsuda index, and an inverse relationship of leg length with systolic blood pressure became apparent. The relationships of sitting height were markedly attenuated by body composition adjustment. The relationships of leg length to sitting height ratio were partially attenuated after adjustment for body composition. Regional body composition measurements (trunk fat and lean mass for sitting height, or leg fat and lean mass for leg length) did not attenuate these associations more than total fat and lean mass (data not shown). These relationships remained similar after adjusting for birthweight and SLI.

For peer review only

For peer review only - http://bmjopen.bmj.com/site/about/guidelines.xhtml

Table 3: Relationship of components of height to CVD risk factors

		Exposure											
		Height (z)			Leg length (z)			Sitting height (z)			Leg length to sitting height (z)		
**Outcome**	**Model**	**B**	**95% CI**	**P**	**B**	**95% CI**	**p**	**B**	**95% CI**	**P**	**B**	**95% CI**	**P**
**Systolic BP (mmHg)**													
	1	0.36	(-0.66, 1.38)	0.49	-0.77	(-1.79, 0.25)	0.14	**1.79**	**(0.79, 2.80)**	**0.001**	**-2.18**	**(-3.18, -1.19)**	**<0.001**
	2	**1.51**	**(0.43, 2.59)**	**0.006**	**-2.19**	**(-3.32, -1.07)**	**<0.001**	**2.87**	**(1.74, 4.01)**	**<0.001**	**-2.80**	**(-3.88, -1.72)**	**<0.001**
	3	-0.38	(-1.69, 0.93)	0.56	**-2.06**	**(-3.20, -0.93)**	**<0.001**	1.04	(-0.21, 2.29)	0.10	**-1.58**	**(-2.67, -0.49)**	**0.005**
**Diastolic BP (mmHg)**													
	1	-0.36	(-1.20, 0.49)	0.41	**-1.11**	**(-1.94, -0.27)**	**0.01**	0.77	(-0.07, 1.62)	0.07	**-1.82**	**(-2.65, -1.00)**	**<0.001**
	2	0.43	(-0.48, 1.34)	0.35	**-1.97**	**(-2.92, -1.02)**	**<0.001**	**1.75**	**(0.80, 2.70)**	**<0.001**	**-2.02**	**(-2.92, -1.11)**	**<0.001**
	3	0.06	(-1.01, 1.13)	0.91	**-1.17**	**(-2.10, -0.24)**	**0.01**	0.84	(-0.18, 1.87)	0.11	**-1.06**	**(-1.96, -1.17)**	**0.24**
**Total Cholesterol (mmol/l)**													
	1	-0.05	(-0.13, 0.04)	0.28	**-0.08**	**(-0.16, 0.00)**	**0.04**	0.02	(-0.06, 0.10)	0.64	**-0.11**	**(-0.19, -0.03)**	**0.009**
	2	0.00	(-0.09, 0.09)	0.99	**-0.12**	**(-0.22, -0.03)**	**0.01**	0.08	(-0.01, 0.17)	0.10	**-0.11**	**(-0.20, -0.02)**	**0.02**
	3	0.04	(-0.06, 0.15)	0.42	-0.08	(-0.17, 0.02)	0.11	0.09	(-0.01, 0.19)	0.08	**-0.09**	**(-0.18, -0.01)**	**0.04**
**Triglycerides (mmol/l)***													
	1	0.01	(-0.03, 0.06)	0.56	-0.02	(-0.06, 0.03)	0.48	**0.05**	**(0.00, 0.09)**	**0.04**	**-0.05**	**(-0.09, -0.01)**	**0.02**
	2	0.04	(-0.01, 0.09)	0.09	**-0.05**	**(-0.10, 0.00)**	**0.05**	**0.07**	**(0.02, 0.12)**	**0.005**	**-0.07**	**(-0.11, -0.02)**	**0.004**
	3	-0.01	(-0.06, 0.05)	0.84	-0.02	(-0.07, 0.02)	0.32	0.01	(-0.04, 0.06)	0.70	-0.02	(-0.06, 0.03)	0.43
**HDL-cholesterol (mmol/l)**													
	1	0.00	(-0.02, 0.02)	0.97	0.01	(-0.02, 0.03)	0.59	-0.01	(-0.03, 0.01)	0.52	0.01	(-0.01, 0.03)	0.23
	2	-0.01	(-0.03, 0.02)	0.63	0.01	(-0.01, 0.04)	0.33	-0.01	(-0.04, 0.01)	0.30	0.02	(-0.01, 0.04)	0.21
	3	0.02	(-0.01, 0.05)	0.13	0.00	(-0.03, 0.03)	0.99	0.02	(-0.01, 0.05)	0.14	-0.01	(-0.04, 0.01)	0.31
**Fasting glucose (mmol/l)**													
	1	-0.06	(-0.12, 0.01)	0.08	**-0.07**	**(-0.13, -0.01)**	**0.02**	-0.01	(-0.08, 0.05)	0.61	**-0.07**	**(-0.13, -0.01)**	**0.02**
	2	-0.03	(-0.10, 0.04)	0.35	**-0.08**	**(-0.15, -0.01)**	**0.02**	0.03	(-0.05, 0.10)	0.48	-0.06	(-0.12, 0.01)	0.09
	3	-0.04	(-0.12, 0.05)	0.42	-0.05	(-0.13, 0.02)	0.17	0.00	(-0.08, 0.08)	0.97	-0.03	(-0.10, 0.05)	0.46

For peer review only - http://bmjopen.bmj.com/site/about/guidelines.xhtml

**120-min glucose (mmol/l)**													
	1	**-0.26**	**(-0.42, -0.10)**	**0.002**	**-0.31**	**(-0.47, -0.15)**	**<0.001**	-0.11	(-0.27, 0.06)	0.20	**-0.27**	**(-0.43, -0.11)**	**0.001**
	2	**-0.18**	**(-0.35, 0.00)**	**0.05**	**-0.34**	**(-0.52, -0.15)**	**<0.001**	0.06	(-0.13, 0.24)	0.54	**-0.20**	**(-0.37, -0.03)**	**0.02**
	3	**-0.24**	**(-0.45, -0.02)**	**0.03**	**-0.19**	**(-0.38, 0.00)**	**0.05**	-0.10	(-0.30, 0.11)	0.36	-0.04	(-0.22, 0.14)	0.70
**Fasting insulin (pmol/l)***													
	1	0.00	(-0.08, 0.07)	0.92	-0.01	(-0.09, 0.06)	0.70	0.01	(-0.06, 0.08)	0.75	-0.03	(-0.10, 0.05)	0.47
	2	0.01	(-0.07, 0.09)	0.79	-0.03	(-0.11, 0.06)	0.53	0.03	(-0.06, 0.11)	0.56	-0.03	(-0.11, 0.05)	0.46
	3	-0.05	(-0.15, 0.04)	0.26	0.04	(-0.04, 0.12)	0.34	-0.08	(-0.17, 0.01)	0.09	0.07	(-0.01, 0.14)	0.10
**HOMA-IR***													
	1	-0.01	(-0.08, 0.06)	0.79	-0.02	(-0.09, 0.05)	0.61	0.01	(-0.07, 0.08)	0.86	-0.03	(-0.10, 0.04)	0.44
	2	0.00	(-0.08, 0.08)	0.96	-0.03	(-0.11, 0.05)	0.48	0.02	(-0.06, 0.11)	0.61	-0.03	(-0.11, 0.05)	0.47
	3	-0.05	(-0.15, 0.04)	0.27	0.04	(-0.04, 0.12)	0.34	-0.08	(-0.17, 0.02)	0.10	0.07	(-0.01, 0.14)	0.10
**HOMA-B**													
	1	-0.11	(-4.95, 4.73)	0.96	-0.93	(-5.76, 3.90)	0.71	1.04	(-3.80, 5.89)	0.67	-1.94	(-6.76, 2.89)	0.43
	2	0.83	(-4.49, 6.15)	0.76	-1.91	(-7.47, 3.65)	0.50	1.99	(-3.59, 7.57)	0.48	-2.28	(-7.58, 3.03)	0.40
	3	-3.59	(-10.1, 2.93)	0.28	1.06	(-4.56, 6.69)	0.71	-4.10	(-10.3, 2.13)	0.20	2.98	(-2.45, 8.40)	0.28
**Insulinogenic index****													
	1	0.04	(-0.06, 0.15)	0.43	0.06	(-0.04, 0.17)	0.25	0.00	(-0.10, 0.11)	0.94	0.07	(-0.04, 0.17)	0.20
	2	0.02	(-0.10, 0.13)	0.77	0.08	(-0.04, 0.20)	0.20	-0.03	(-0.16, 0.09)	0.58	0.06	(-0.05, 0.18)	0.29
	3	0.05	(-0.10, 0.20)	0.54	0.11	(-0.01, 0.24)	0.08	-0.03	(-0.17, 0.11)	0.68	0.08	(-0.05, 0.20)	0.22
**Matsuda index***													
	1	**0.08**	**(0.01, 0.16)**	**0.04**	**0.11**	**(0.03, 0.18)**	**0.005**	0.02	(-0.06, 0.09)	0.62	**0.11**	**(0.03, 0.18)**	**0.005**
	2	0.04	(-0.04, 0.13)	0.29	**0.13**	**(0.04, 0.21)**	**0.004**	-0.04	(-0.12, 0.04)	0.32	**0.09**	**(0.01, 0.17)**	**0.03**
	3	0.05	(-0.05, 0.14)	0.32	0.02	(-0.06, 0.10)	0.56	0.03	(-0.06, 0.12)	0.53	-0.01	(-0.08, 0.07)	0.88
**Disposition index**													
	1	0.01	(-0.58, 0.60)	0.96	0.16	(-0.43, 0.74)	0.61	-0.18	(-0.77, 0.41)	0.55	0.32	(-0.27, 0.90)	0.29
	2	-0.14	(-0.79, 0.51)	0.67	0.32	(-0.36, 0.99)	0.35	-0.34	(-1.01, 0.34)	0.33	0.37	(-0.27, 1.02)	0.25
	3	0.36	(-0.48, 1.20)	0.40	0.14	(-0.58, 0.87)	0.70	0.24	(-0.56, 1.04)	0.55	-0.08	(-0.77, 0.62)	0.83

BP, blood pressure. * Outcome variables were log-transformed to ensure normality. ** Outcome variable was Fisher-Yates transformed to ensure normality Components of height were z-standardised. Model 1 was adjusted for age, sex and interaction of age and sex. Model 2 was adjusted for age, sex, interaction of age and sex, leg length and sitting height when the exposure of interest were leg length or sitting height, and for age, sex, interaction of age and sex, standing height and leg length to sitting height ration when the exposures of interest were standing height and leg length to sitting height ratio. Model 3 was built based on Model 2 and additionally adjusted for lean mass, fat mass, interaction of sex and lean mass, and interaction of sex and fat mass.

For peer review only - http://bmjopen.bmj.com/site/about/guidelines.xhtml

Relationships of CVD risk factors with leg length and sitting height simultaneously

The associations of leg length and sitting height simultaneously with CVD risk factors are shown in Table 3, and as contour plots in Figure 1 and Supplementary Figures 1 and 2. Because of the opposing relationships of leg length and sitting height with CVD risk factors, these associations tended to be accentuated. In general, at any leg length, greater sitting height was associated with a more adverse profile; at any sitting height, greater leg length was associated with a more favourable profile. The healthiest profiles tended to be in people with long legs and short sitting height. However, there was variation in patterns. For lean mass, systolic blood pressure, triglycerides, HDL-cholesterol, and fasting glucose (contour lines near-horizontal), the associations with sitting height were stronger than those with leg length (Figure 1). For 120-minute glucose and Matsuda index (contour lines near-vertical), the associations were stronger with leg length. For BMI, fat mass, total cholesterol and diastolic blood pressure (contour lines near-diagonal), the associations with leg length and sitting height, though in opposite directions, were of similar strength. There were no relationships with HOMA-IR, HOMA-B, Insulinogenic index or Disposition index.

Relationships with parental height and intergenerational change in height:

There were no relationships between height of either parent or mid-parental height with offspring CVD risk factors (Table 4). A greater increase in leg length between generations was associated with lower BMI, fasting insulin, and HOMA-IR while a greater increase in sitting height was associated with higher BMI. There were no interactions between parental and offspring heights on offspring CVD risk factors.

For peer review only - http://bmjopen.bmj.com/site/about/guidelines.xhtml

Table 4: Relationship of Parental height, and its components and intergenerational change in height with offspring CVD parameters at 21 years

Outcome at 21 years	Mid-parental height	Father's Height	Father's Leg length	Father's Sitting height	Mother's Height	Mother's Leg length	Mother's Sitting height	Intergenerational change in height	Intergenerational change in leg length	Intergenerational change in sitting height
BMI	0.07	0.03	-0.03	0.09	0.00	-0.04	0.05	-0.02	-0.13*	0.16**
SLI score	0.15**	0.13*	0.04	0.18**	0.10	0.07	0.10	0.00	0.05	-0.04
Systolic BP	0.07	0.04	-0.05	0.13*	0.06	0.01	0.10	-0.00	-0.09	0.03
Diastolic BP	0.02	0.02	-0.02	0.08	-0.04	-0.09	0.05	0.05	-0.09	0.03
Cholesterol	0.01	-0.03	-0.11	0.08	0.02	0.002	0.04	-0.06	-0.07	-0.01
Triglycerides	0.08	0.01	-0.01	0.04	0.04	-0.00	0.08	0.02	-0.03	0.06
HDL	-0.01	-0.02	-0.04	0.02	0.05	0.06	0.02	-0.01	0.02	-0.02
Fasting glucose	-0.04	-0.10	-0.08	-0.09	-0.00	-0.03	0.03	-0.06	-0.09	0.01
120-min glucose	-0.07	-0.10	-0.13*	-0.03	-0.05	-0.07	-0.01	-0.09	-0.09	-0.02
Fasting insulin	0.00	0.01	0.04	-0.03	0.01	-0.02	0.03	-0.09	-0.12*	-0.00
120-min insulin	0.02	-0.04	-0.05	-0.01	0.03	-0.04	0.09	-0.03	-0.05	0.02
HOMA-IR	0.00	0.00	0.04	-0.04	0.01	-0.02	0.04	-0.10	-0.13*	-0.00
HOMA-B	0.02	0.03	0.05	0.00	0.01	0.01	-0.01	-0.04	-0.06	0.04
Insulinogenic Index	0.11	0.11	0.13	0.03	0.05	0.05	0.03	-0.10	-0.03	-0.03
Matsuda Index	0.04	0.10	0.07	0.09	-0.01	0.01	-0.02	0.05	0.08	-0.05
Disposition Index	0.06	0.10	0.10	0.07	0.02	0.02	0.02	-0.07	0.00	-0.10

Values are Pearson correlation coefficients adjusted for age and gender, as appropriate; exposures and outcomes are Z-standardized.
*p<0.05, ** p<0.01,*** p<0.001

For peer review only

Discussion

There was an intergenerational increase in total height, leg length and sitting height in this young adult Indian urban cohort. Greater total adult height and leg length were associated with lower BMI, greater lean body mass, and lower CVD risk factors, while a greater sitting height was associated with higher BMI, lean and fat mass and higher CVD risk factors. These associations were accentuated when leg length and sitting height were examined simultaneously, and leg length to sitting height ratio was strongly negatively related to CVD risk factors. The relationships with sitting height were greatly attenuated by adjusting for lean and fat mass. Parental height was unrelated to offspring CVD risk factors; however a greater intergenerational increase in leg length was associated with lower insulin and insulin resistance.

The associations of shorter leg length with higher CVD risk factors in our study are consistent with findings from high-income countries which have shown inverse associations of leg length with blood pressure, glucose intolerance and dyslipidaemia[11-14]. The associations with sitting height are less consistent, with some studies showing positive associations with CVD risk factors (higher insulin resistance, lower HDL-cholesterol) and some showing no associations[11,12,14]. Shorter leg length has been associated with higher LDL-cholesterol, and greater sitting height with higher diabetes risk and dyslipidaemia in China[30]. Other studies in LMICs have shown no relationships between height components and CVD risk[5,6].

Similar to our findings, longer leg length has been associated with lower BMI, and greater sitting height with higher BMI[15,16]. No previous studies have examined associations between components of adult height and CVD risk in relation to direct measurements of fat and lean mass. While longer leg length was associated with greater lean mass and lower body fat percent, this did not appear to explain the inverse association between leg length and CVD risk factors – only the relationship between leg length and Matsuda index was attenuated after

For peer review only - http://bmjopen.bmj.com/site/about/guidelines.xhtml

adjustment for lean and fat mass. In contrast, our data indicate that the relationship between sitting height and CVD risk factors could be partly explained by higher fat mass.

Secular increases in height in high-income countries are now slowing down[31]. Much of this increase is reflected in greater leg length[7]; this increment has been shown to occur by 2 years of age[7,32,33]. Leg length increases faster than sitting height in infancy suggesting that it may be related to better fetal and/or infant health and nutrition[34]. In contrast, sitting height increases more than leg length in later childhood,[32-34] due to faster growth of vertebrae than long bones during the pubertal growth spurt[15,32,33]. However, direct evidence that leg length and sitting height reflect conditions in early life and later childhood respectively is lacking. Birth weight, which might be expected to predict longer leg length, is equally strongly predictive of sitting height,[11,14] as in our study. The independent relationships of leg length and sitting height with CVD risk factors suggest that patterns and determinants of linear growth are important and may influence body composition and CVD risk.

Our data do not elucidate the mechanisms linking greater sitting height to increased adiposity or greater leg length to lower CVD risk. Lower birth and infant weight are associated with lower adult lean mass, while greater weight gain in later childhood is associated with increased adult adiposity in several countries including LMICs[35]. Both are associated with a higher prevalence of CVD, type 2 diabetes and their risk factors[36,37]. These findings led to the developmental programming hypothesis that early undernutrition permanently alters the body's structure and metabolism leaving an increased vulnerability to the adverse effects of 'excess' nutrition in later life[38]. This sequence has been suggested as a possible explanation for the rising epidemics of CVD and diabetes, and high risk factor levels (as exemplified by our cohort), in LMICs. If it is true that lower leg length reflects poorer fetal and infant environment, reduced lean body mass, and adverse metabolic programming, and greater sitting height reflects a better childhood

For peer review only - http://bmjopen.bmj.com/site/about/guidelines.xhtml

environment, increased adiposity and greater pubertal growth, our findings linking CVD risk factors to these height components would be broadly consistent with this hypothesis. Our findings do not suggest that the relationship between short height and CVD risk reflects lower socio-economic status. Firstly, we found no relationships of socio-economic status with CVD risk factors. Secondly, we would expect both short leg length and short sitting height to be associated with higher CVD risk. Although genetic explanations for the associations between components of height and CVD risk factors cannot be ruled out, common genes that influence both height and CVD have not been identified. We have not explored genetic determinants in our study.

A greater increase in leg length between generations was associated with lower fasting insulin and insulin resistance. No previous studies have examined changes in leg length or sitting height between generations. A study in Scotland showed that although short height of both parents was independently associated with increased risk of coronary heart disease in the offspring, the association was stronger with mothers' height[39]. A study from India showed that shorter maternal but not paternal height was associated with higher risk of glucose intolerance in the offspring[40]. A greater increase in leg length between generations may reflect better fetal and infant growth and this may have persisting effects on offspring development and health. This may also be reflected through greater insulin sensitivity promoting greater growth.

The PCS is one of few cohorts in a LMIC with follow-up from childhood to adult life and measurements of adult height components and parental heights. Our loss to follow up of ~25% is low for a long term longitudinal study and adds to internal validity. Moreover our study sample was similar in key characteristics to those who were lost to follow up. Limitations were a lack of data on length, and on height components at birth and longitudinally throughout the growing

For peer review only - http://bmjopen.bmj.com/site/about/guidelines.xhtml

period. Although the participants were born in one hospital in Pune, it is the second largest in Pune and offers obstetric services to a wide range of socio-economic classes.

Conclusions: Ours is the first study to examine associations of CVD risk factors with components of adult height, using body composition measures to understand the relationship. Shorter leg length and greater sitting height were associated with a more adverse CVD risk factor profile. We speculate that the associations with leg length and sitting height reflect the benefits of better growth in early childhood which is protective, and accelerated growth in later childhood which may be detrimental, and that these relationships are partially explained by body composition. Perhaps the strongest message that emerges from this analysis is the need to gain a better understanding of the determinants of height components.

Acknowledgments

We are grateful to the study participants for taking part in this study. We thank Dr KJ Coyaji, Medical Director of the KEM Hospital and Dr VS Padbidri, Director Research for providing research facilities. We thank Mrs PC Yajnik, Ms LV Ramdas, Ms Sonali Hardikar, Ms Sonali Wagle, Ms.Vaishali Deshpande, Ms Nilam Memane, Ms Deepa Raut, Ms Pallavi Hardikar, Ms Sonal Kasture, Ms Vaishali Kantikar, Ms Komal Advani, Ms Neha Gurav, Ms Rupali Saswade and Mr Arjunsing Patilkendre for their contribution in collection of the data. We also thank Mr TM Deokar, Mr SD Chougule, Mr AB Gaikwad, Mr ML Hoge, Mr. Vipul Wagh, Mr SN Khemkar, Mr SB Wagh, Mr BS Jadhav and Ms Prachi Katre for their invaluable contribution to the study. Dr Sharad Gore helped with statistical queries. We also acknowledge the support of Sneha-India.

Funding

The Study is funded by The Wellcome Trust, UK (Grant Number: 083460/Z/07/Z), the Medical Research Council, UK and the Department for International Development, UK.

For peer review only - http://bmjopen.bmj.com/site/about/guidelines.xhtml

Conflict of interest

None of the authors had any financial or personal conflicts of interest associated with this manuscript.

Contributor statement

CSY and CHDF designed the study, analysed and interpreted the data and wrote the manuscript. KK, SMJ and CDG analysed and interpreted the data, and wrote the manuscript. HL, DSB, CJ and ASN carried out data collection and critically reviewed the manuscript. CO analysed and interpreted the data, and critically reviewed the manuscript. AB, SAB and ANP designed the study and critically reviewed the manuscript. All authors approved the final version. CSY is the guarantor of this work and as such, had full access to all the data in the study and takes responsibility of the integrity of the data and the accuracy of the data analysis.

For peer review only

References:

1. The Emerging Risk Factors Collaboration. Adult height and the risk of cause-specific death and vascular morbidity in 1 million people: individual participant meta-analysis. *Int J Epidemiol* 2012;**41**:1419-33.
2. Davey Smith G, Hart C, Upton M et al. Height and risk of men among men and women: aetiological implications of associations with cardiovascular disease and cancer mortality. *J Epidemiol Community Health* 2000;**54**:97-103.
3. Paajanen TA, Oksala NKJ, Kuukasjarvi P et al. Short stature is associated with coronary heart disease: a systematic review of the lierature and a meta-analysis. *Eur Heart J* 2010;**31**:1802-1809.
4. Man Ying Lee C, Barzi F, Woodward M et al. Adult height and the risks of cardiovascular disease and major causes of death in the Asia-Pacific region: 21000 deaths in 510000 men and women. *Int J Epidemiol* 2009;**38**:1060-1071.
5. Song YM, Davey Smith G, Sung J. Adult height and cause- specific mortality: a large cohort study of South Korean men. *Am J Epidemiol* 2003;**158**:479-485.
6. Sichieri R, Siqueira KS, Pereira RA et al. Short stature and hypertension in the city of Rio de Janeriro, Brazil. *Public Health Nutr* 2003;**3**:77-82.
7. Cole TJ. Secular trends in growth. *Proc Nutr Soc* 2000;**59**:317-24.
8. Sorensen HT, Sabroe S, Rothman KJ et al. Birth weight and length as predictors for adult height. *Am J Epidemiol* 1999;**149**:726-729.
9. Barker DJP, Osmond C, Winter PD et al. Weight in infancy and death from ischaemic heart disease. *Lancet* 1989;**ii**:577-80.
10. Marmot MG, Shipley MJ, Rose G. Inequalities in death – specific explanations of a general pattern? *Lancet* 1984;**i**:1003-6.
11. Davey Smith G, Greenwood R, Gunnell D et al. Leg length, insulin resistance and coronary heart disease: The Caerphilly Study. *J Epidemiol Community Health* 2001;**55**:867-872.
12. Lawlor DA, Taylor M, Davey Smith G et al. Associations of components of adult height with coronary heart disease in postmenopausal women: The British Women's Heart and Health Study. *Heart* 2004;**90**:745-749.
13. Whitley E, Martin RM, Davey Smith G et al. The association of childhood height, leg length and other measures of skeletal growth with adult cardiovascular disease: the Boyd-Orr cohort. *J Epidemiol Community Health* 2012;**66**:18-23.

For peer review only - http://bmjopen.bmj.com/site/about/guidelines.xhtml

14. Lawlor DA, Ebrahim S, Davey Smith G. The association between components of adult height and type II diabetes and insulin resistance: the British Women's Heart and Health Study. *Diabetologia* 2002;**45**:1097-1106.
15. Lorentzon M, Norjavaara E, Kindblom JM. Pubertal timing predicts leg length and childhood body mass index predicts sitting height in young adult men. *J Pediatr* 2011;**158**:452-7.
16. Velasquez-Melendez G, Silveira EA, Allencastro-Souza P et al. Relationship between sitting-height-to-stature ratio and adiposity in Brazilian women. *Ann Hum Biol* 2005;**17**:646-53.
17. Bavdekar A, Yajnik CS, Fall CH et al. Insulin resistance syndrome in 8-year-old Indian children: small at birth, big at 8 years, or both? *Diabetes* 1999;**48**:2422-2429.
18. Freeman JV, Cole TJ, Chinn S et al. Cross-sectional stature and weight reference curves for the UK, 1990. *Arch Dis Child* 1995;**73**:17-24.
19. Definition, Diagnosis and Classification of Diabetes Mellitus and its Complications Report of a WHO Consultation. Part 1: Diagnosis and Classification of Diabetes Mellitus. Geneva: World Health Organization; 1999.
20. International Institute for Population Sciences and Macro International. National Family Health Survey (NHFS-3), India 2005-06. Mumbai: IIPS; 2007.
21. The Oxford Centre for Diabetes, Endocrinology and Metabolism, Diabetes Trials Unit. HOMA calculator. Available from http://www.dtu.ox.ac.uk, accessed 1 July 2014.
22. Wareham NJ, Phillips DI, Byrne CD et al. The 30 minute insulin incremental response in an oral glucose tolerance test as a measure of insulin secretion. *Diabet Med* 1995;**12**:931.
23. DeFronzo RA, Matsuda M. Reduced time points to calculate the composite index. *Diabetes Care* 2010;**33**:e93
24. Bergman RN, Phillips LS, Cobelli C. Physiologic evaluation of factors controlling glucose tolerance in man. Measurement of insulin sensitivity and β-cell glucose sensitivity from the response to intravenous glucose. *J Clin Invest* 1981;68:1456–67.
25. BMI classification. World Health Organization. Global database on body mass index.Available from http://www.who.int/bmi, accessed 15 July 2014.
26. Centre for Disease Control and Prevention. www.cdc.gov/growthcharts/zscore.htm, accessed 15 July 2014.
27. American Diabetes Association. Standards of medical care in diabetes – 2013. *Diabetes Care* 2013;**36**:S11-66.

For peer review only - http://bmjopen.bmj.com/site/about/guidelines.xhtml

28. National Cholesterol Education Program Expert Panel on Detection, Evaluationand Treatment of High Blood Cholesterol in Adults (Adult Treatment Panel III). 2002. (Publication No. NIH 02:5215).
29. Alberti KG, Zimmet P, Shaw J. Metabolic syndrome - a new world-wide definition. A Consensus Statement from the International Diabetes Federation. *Diabet Med.* 2006;**23**:469-480.
30. Schooling CM, Jiang C, Lam TH et al. Height, its components and cardiovascular risk among older Chinese: a cross-sectional analysis of the Guangzhou Biobank Cohort Study. *Am J Public Health* 2007;**97**:1834-1841.
31. Larnkjaer A, Schroder SA, Schmidt IM et al. Secular change in adult stature has come to a halt in northern Europe and Italy. *Acta Paediatr* 2006;**96**:754-755.
32. Tanner JM, Hayashi T, Preece MA et al. Increase in growth of leg relative to trunk in Japanese children and adults from 1957 to 1977: comparison with British and with Japanese Americans. *Ann Hum Biol* 1982;**9**:411-23.
33. Cameron N, Tanner JM, Whitehouse RH. A longitudinal analysis of the growth of limb segments in adolescence. *Ann Hum Biol* 1982;**9**:211-20.
34. Gunnell D. Commentary: Early insights into height, leg length, proportionate growth and health. *Int J Epidemiol* 2001;**30**:221-2.
35. Kuzawa CW, Hallal PC, Adair L at al. Birth weight, postnatal weight gain, and adult body composition in five low and middle income countries. *Am J Hum Biol* 2012;**24**:5-13.
36. Eriksson JG, Forsen T, Tuomilehto HJ et al. Early growth and coronary heart disease in later life: longitudinal study. *BMJ* 2001;**322**:949-53.
37. Bhargava SK, Sachdev HPS, Fall CHD et al. Relation of serial changes in childhood body mass index to impaired glucose tolerance in young adulthood. *New England J Med* 2004;**350**:865-75.
38. Barker DJP, Gluckman PD, Godfrey KM et al. Fetal nutrition and cardiovascular disease in adult life. *Lancet*1993;**341**:938-41.
39. Gray L, Davey Smith G, McConnachie A et al. Parental height in relation to offspring coronary heart disease: examining transgenerational influences on health using the west of Scotland Midspan Family Study. *Int J Epidemiol* 2012;**41**:1776-1785.
40. Raghupathy R, Antonisamy B, Geethanjali FS et al. Glucose tolerance, insulin resistance and insulin secretio in young south Indian adults: relationship to parental size, neonatal size and childhood body mass index. *Diab Res Clin Pract* 2010;**87**:283-92.

For peer review only - http://bmjopen.bmj.com/site/about/guidelines.xhtml

Figure 1: Relationships of leg length and sitting height with selected CVD risk factors

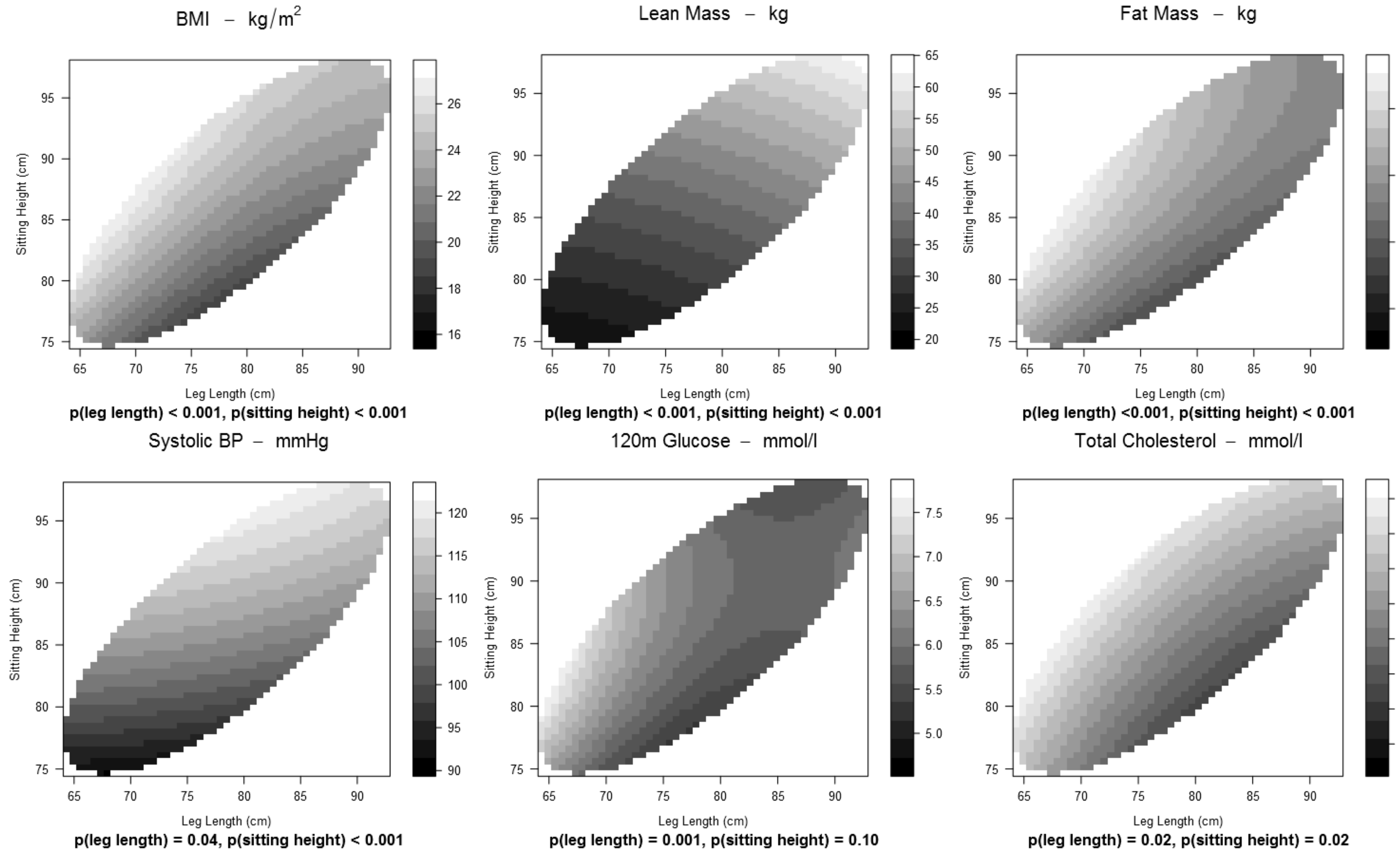

Supplementary Figure 1: Relationships of leg length and sitting height with selected CVD risk factors

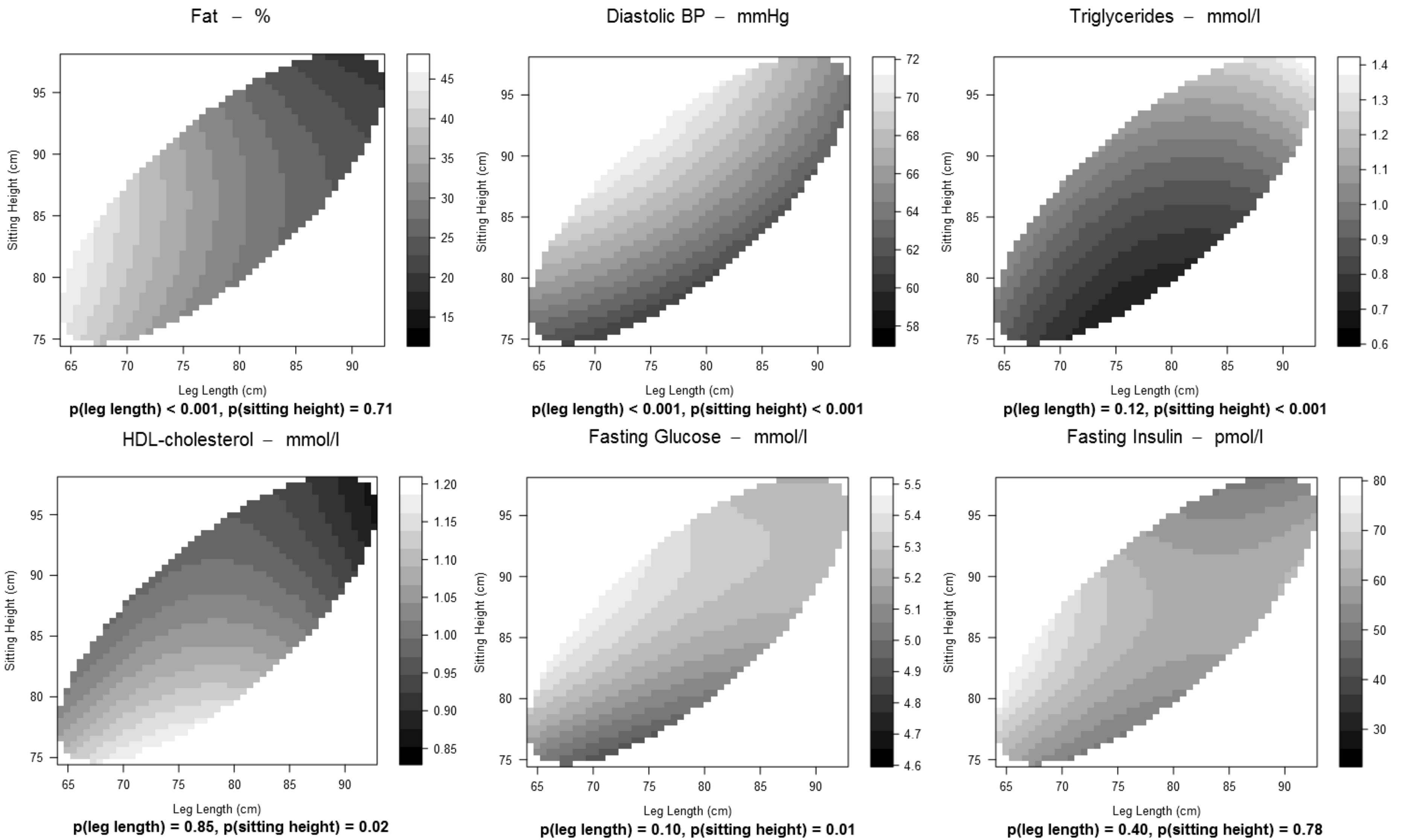

Supplementary Figure 2: Relationships of leg length and sitting height with selected CVD risk factors

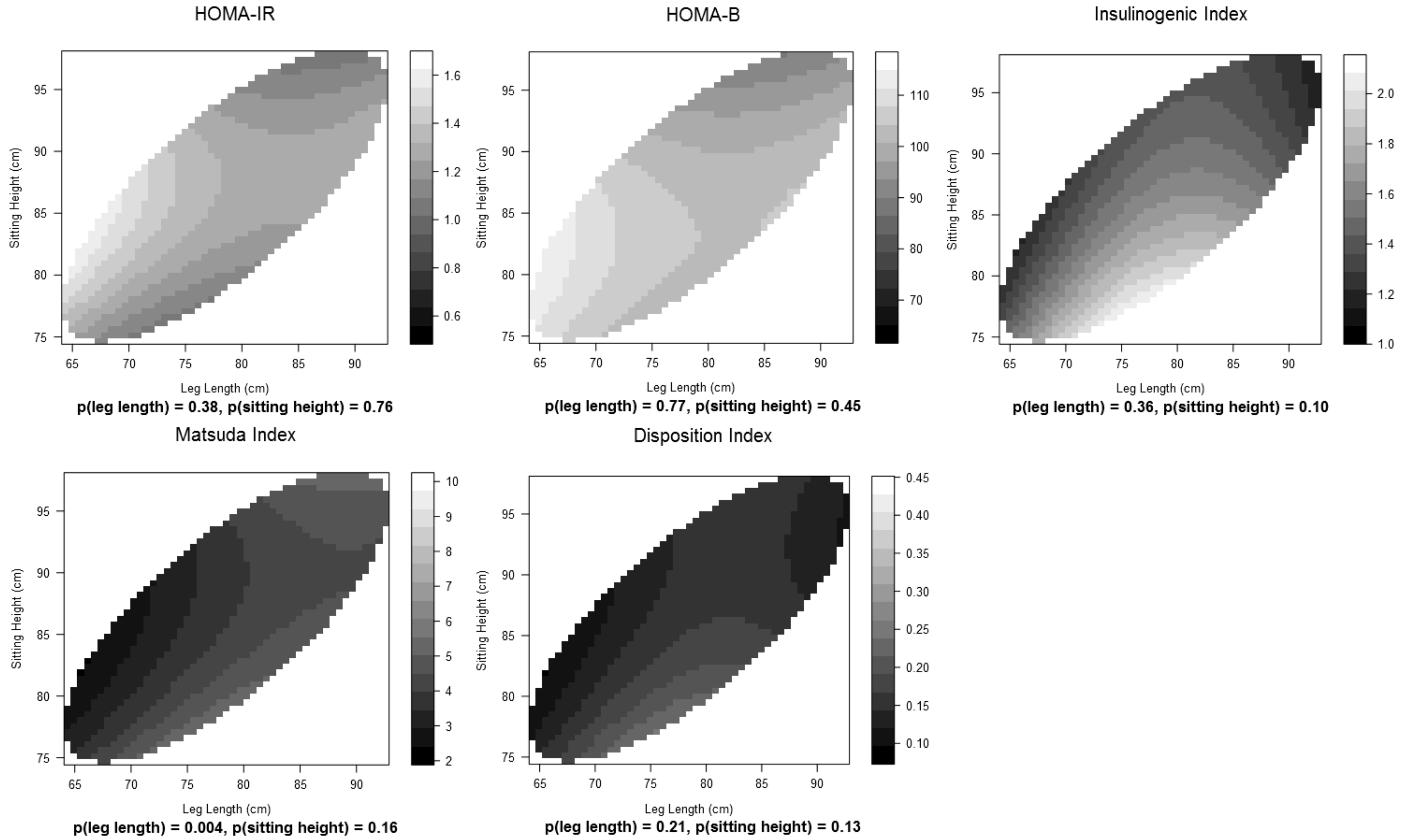

STROBE Statement—checklist of items that should be included in reports of observational studies

	Item No	Recommendation	Page number
**Title and abstract**	1	(*a*) Indicate the study's design with a commonly used term in the title or the abstract	2
		(*b*) Provide in the abstract an informative and balanced summary of what was done and what was found	2
**Introduction**			
Background/rationale	2	Explain the scientific background and rationale for the investigation being reported	4
Objectives	3	State specific objectives, including any prespecified hypotheses	4
**Methods**			
Study design	4	Present key elements of study design early in the paper	5
Setting	5	Describe the setting, locations, and relevant dates, including periods of recruitment, exposure, follow-up, and data collection	5-6
Participants	6	(*a*) *Cohort study*—Give the eligibility criteria, and the sources and methods of selection of participants. Describe methods of follow-up<br>*Case-control study*—Give the eligibility criteria, and the sources and methods of case ascertainment and control selection. Give the rationale for the choice of cases and controls<br>*Cross-sectional study*—Give the eligibility criteria, and the sources and methods of selection of participants	5
		(*b*) *Cohort study*—For matched studies, give matching criteria and number of exposed and unexposed<br>*Case-control study*—For matched studies, give matching criteria and the number of controls per case	
Variables	7	Clearly define all outcomes, exposures, predictors, potential confounders, and effect modifiers. Give diagnostic criteria, if applicable	5-6
Data sources/ measurement	8*	For each variable of interest, give sources of data and details of methods of assessment (measurement). Describe comparability of assessment methods if there is more than one group	5-6
Bias	9	Describe any efforts to address potential sources of bias	6
Study size	10	Explain how the study size was arrived at	5
Quantitative variables	11	Explain how quantitative variables were handled in the analyses. If applicable, describe which groupings were chosen and why	6-7
Statistical methods	12	(*a*) Describe all statistical methods, including those used to control for confounding	6-7
		(*b*) Describe any methods used to examine subgroups and interactions	7
		(*c*) Explain how missing data were addressed	7
		(*d*) *Cohort study*—If applicable, explain how loss to follow-up was addressed<br>*Case-control study*—If applicable, explain how matching of cases and controls was addressed<br>*Cross-sectional study*—If applicable, describe analytical methods taking account of sampling strategy	
		(*e*) Describe any sensitivity analyses	7-8

Continued on next page

For peer review only - http://bmjopen.bmj.com/site/about/guidelines.xhtml

**Results**			
Participants	13*	(a) Report numbers of individuals at each stage of study—eg numbers potentially eligible, examined for eligibility, confirmed eligible, included in the study, completing follow-up, and analysed	7
		(b) Give reasons for non-participation at each stage	7
		(c) Consider use of a flow diagram	
Descriptive data	14*	(a) Give characteristics of study participants (eg demographic, clinical, social) and information on exposures and potential confounders	7-8
		(b) Indicate number of participants with missing data for each variable of interest	8
		(c) *Cohort study*—Summarise follow-up time (eg, average and total amount)	
Outcome data	15*	*Cohort study*—Report numbers of outcome events or summary measures over time	8-9
		Case-control study—Report numbers in each exposure category, or summary measures of exposure	
		Cross-sectional study—Report numbers of outcome events or summary measures	
Main results	16	(*a*) Give unadjusted estimates and, if applicable, confounder-adjusted estimates and their precision (eg, 95% confidence interval). Make clear which confounders were adjusted for and why they were included	8-15
		(*b*) Report category boundaries when continuous variables were categorized	
		(*c*) If relevant, consider translating estimates of relative risk into absolute risk for a meaningful time period	
Other analyses	17	Report other analyses done—eg analyses of subgroups and interactions, and sensitivity analyses	8-15
**Discussion**			
Key results	18	Summarise key results with reference to study objectives	16
Limitations	19	Discuss limitations of the study, taking into account sources of potential bias or imprecision. Discuss both direction and magnitude of any potential bias	18
Interpretation	20	Give a cautious overall interpretation of results considering objectives, limitations, multiplicity of analyses, results from similar studies, and other relevant evidence	19
Generalisability	21	Discuss the generalisability (external validity) of the study results	18-19
**Other information**			
Funding	22	Give the source of funding and the role of the funders for the present study and, if applicable, for the original study on which the present article is based	19

*Give information separately for cases and controls in case-control studies and, if applicable, for exposed and unexposed groups in cohort and cross-sectional studies.

Note: An Explanation and Elaboration article discusses each checklist item and gives methodological background and published examples of transparent reporting. The STROBE checklist is best used in conjunction with this article (freely available on the Web sites of PLoS Medicine at http://www.plosmedicine.org/, Annals of Internal Medicine at http://www.annals.org/, and Epidemiology at http://www.epidem.com/). Information on the STROBE Initiative is available at www.strobe-statement.org.

For peer review only

For peer review only - http://bmjopen.bmj.com/site/about/guidelines.xhtml

DO COMPONENTS OF ADULT HEIGHT PREDICT BODY COMPOSITION AND CARDIOMETABOLIC RISK IN A YOUNG ADULT SOUTH ASIAN INDIAN POPULATION? FINDINGS FROM A HOSPITAL-BASED COHORT STUDY IN PUNE, INDIA: THE PUNE CHILDREN’S STUDY.

Journal:	*BMJ Open*
Manuscript ID	bmjopen-2020-036897.R1
Article Type:	Original research
Date Submitted by the Author:	16-Jul-2020
Complete List of Authors:	Kumaran, Kalyanaraman; University of Southampton, MRC Lifecourse Epidemiology Unit; CSI Holdsworth Memorial Hospital, Epidemiology Research Unit<br>Joshi, Suyog; KEM Hospital Pune, Diabetes Unit<br>Di Gravio, Chiara; Vanderbilt University<br>Lubree, Himangi; KEM Hospital Pune, Vadu Rural Health Centre<br>Joglekar, Charudatta ; BKL Walawalkar Rural Medical College, Regional Centre for Adolescent Health and Nutrition<br>Bhat, Dattatray; KEM Hospital Pune, Diabetes Unit<br>Kinare, Arun; Bharati Vidyapeeth Deemed University Medical College, Department of Radiology<br>Bavdekar, Ashish; KEM Hospital Pune, Department of Paediatrics<br>Bhave, Sheila; KEM Hospital Pune, Department of Paediatrics<br>Pandit, Anand; KEM Hospital Pune, Department of Paediatrics<br>Osmond, Clive; University of Southampton,<br>Yajnik, Chittaranjan; KEM Hospital Pune, Diabetes Unit<br>Fall, Caroline ; University of Southampton, MRC Lifecourse Epidemiology Unit
**Primary Subject Heading**:	Epidemiology
Secondary Subject Heading:	Public health
Keywords:	Cardiac Epidemiology < CARDIOLOGY, EPIDEMIOLOGY, DIABETES & ENDOCRINOLOGY, PREVENTIVE MEDICINE

SCHOLARONE™ Manuscripts

For peer review only - http://bmjopen.bmj.com/site/about/guidelines.xhtml

I, the Submitting Author has the right to grant and does grant on behalf of all authors of the Work (as defined in the below author licence), an exclusive licence and/or a non-exclusive licence for contributions from authors who are: i) UK Crown employees; ii) where BMJ has agreed a CC-BY licence shall apply, and/or iii) in accordance with the terms applicable for US Federal Government officers or employees acting as part of their official duties; on a worldwide, perpetual, irrevocable, royalty-free basis to BMJ Publishing Group Ltd ("BMJ") its licensees and where the relevant Journal is co-owned by BMJ to the co-owners of the Journal, to publish the Work in this journal and any other BMJ products and to exploit all rights, as set out in our licence.

The Submitting Author accepts and understands that any supply made under these terms is made by BMJ to the Submitting Author unless you are acting as an employee on behalf of your employer or a postgraduate student of an affiliated institution which is paying any applicable article publishing charge ("APC") for Open Access articles. Where the Submitting Author wishes to make the Work available on an Open Access basis (and intends to pay the relevant APC), the terms of reuse of such Open Access shall be governed by a Creative Commons licence – details of these licences and which Creative Commons licence will apply to this Work are set out in our licence referred to above.

Other than as permitted in any relevant BMJ Author's Self Archiving Policies, I confirm this Work has not been accepted for publication elsewhere, is not being considered for publication elsewhere and does not duplicate material already published. I confirm all authors consent to publication of this Work and authorise the granting of this licence.

For peer review only

DO COMPONENTS OF ADULT HEIGHT PREDICT BODY COMPOSITION AND CARDIOMETABOLIC RISK IN A YOUNG ADULT SOUTH ASIAN INDIAN POPULATION? FINDINGS FROM A HOSPITAL-BASED COHORT STUDY IN PUNE, INDIA: THE PUNE CHILDREN'S STUDY.

Kumaran K[1,2], Joshi SM[3], Di Gravio C[1], Lubree H[4], Joglekar C[5], Bhat DS[3], Kinare AS[3], Bavdekar A[4], Bhave SA[6], Pandit AN[6], Osmond C[1], Yajnik CS[3], Fall CHD[1]

[1]MRC Lifecourse Epidemiology Unit, University of Southampton, Southampton, UK.

[2]Epidemiology Research Unit, CSI Holdsworth Memorial Hospital, Mysore, India

[3]Diabetes Unit, KEM Hospital Research Centre, Pune, India.

[4]Vadu Rural Health Program, KEM Hospital Research Centre, Pune, India

[5]Department of Statistics, BKL Walawalkar Hospital and Diagnostic Centre, Ratnagiri, India

[6]Department of Pediatrics, KEM Hospital, Pune, India.

Corresponding author: Chittaranjan S. Yajnik, Kamalnayan Bajaj Diabetology Research Centre, Diabetes Unit, King Edward Memorial Hospital Research Centre, Rasta Peth, Pune 411011, India

Telephone: +91-20-26061958
Fax: +91-20-26111958
Email: diabetes@kemdiabetes.org; csyajnik@gmail.com

WORD COUNT ABSTRACT: 233

WORD COUNT MAIN TEXT: 2989

For peer review only

Abstract

Objectives: We investigated whether the relationship between components of height and CVD risk may be explained by body composition. We also examined relationships between parental heights and offspring CVD risk.

Design: A cohort study using cross-sectional data.

Setting: A secondary care hospital setting in Pune, India

Participants: We studied 357 young adults and their parents in the Pune Children's Study.

Primary and secondary outcomes: We measured weight, total height, leg length, sitting height, plasma glucose, insulin and lipids, and blood pressure. Total and regional lean and fat mass were measured by dual x-ray absorptiometry.

Results: Leg length was inversely, and sitting height directly related to BMI. Total height and leg length were directly related to lean mass while sitting height was directly related to both lean and fat mass. Leg length was inversely related to systolic blood pressure and 120-minute glucose, independent of lean and fat mass. Sitting height was directly related to systolic blood pressure and triglycerides; these relationships were attenuated on adjustment for lean and fat mass. When examined simultaneously, greater leg length was protective and greater sitting height was associated with a more detrimental CVD risk profile.

Conclusions: Shorter adult leg length and greater sitting height are associated with a more adverse CVD risk factor profile. The mechanisms need further study but our findings suggest a role for lean and fat mass.

Key words: adult height; leg length; sitting height; intergenerational change; cardiometabolic risk factors; India

For peer review only - http://bmjopen.bmj.com/site/about/guidelines.xhtml

Strength and limitations of the study:

- One of few birth cohorts in a low- and middle-income country setting with data on parental and offspring height, and height components
- Data available on body composition measurements using DXA
- Glucose-insulin measurements available as part of OGTT
- Participants born in one hospital in Pune, India
- ~25% loss to follow-up between childhood and young adulthood.

For peer review only

For peer review only - http://bmjopen.bmj.com/site/about/guidelines.xhtml

Introduction

Short adult height is associated with an increased risk of cardiovascular disease (CVD) and its risk factors in both high-income and low- and middle-income countries (LMICs),[1-4] although the association is less consistent in the latter[5,6].The reasons for the association remain unknown. Intrauterine and infant growth are determinants of final height[7,8]. Impaired fetal and infant growth are also associated with CVD[9]. It is therefore possible that the association between height and CVD is related to early life programming. Short height may also reflect lower socio-economic class,[10] and deprivation throughout childhood. The relationship between height and CVD may have a genetic basis; height is partly genetically determined as are some CVD risk factors. Finally, 'reverse causality' (i.e. common risk factors for osteoporosis and CVD which may lead to height shrinkage) has been suggested as a possible reason for the relationship between height and CVD[11].

The components of height i.e. leg length and sitting height have shown independent and contrasting relationships with CVD risk factors. Longer leg length has been associated with lower CVD risk,[12-17], while greater sitting height has shown either no association,[12,13] or an association with higher risk[15]. Most of these studies have been carried out in high-income countries. However the reasons for these differential associations remain speculative. It has been suggested that during intrauterine insults, blood flow to the brain is selectively preserved at the expense of the peripheral organs including limbs ('brain sparing effect')[18]. This may result in shorter legs. It has also been suggested that increases in leg length occur mainly during infancy and childhood reflecting better nutrition and environmental circumstances during early life[18,19]. These factors might explain the relationship between leg length and CVD risk. In contrast, sitting height increases mainly during the pubertal growth spurt when the trunk and vertebral bones continue to grow after the limbs have stopped growing[19]. Accelerated pubertal

For peer review only - http://bmjopen.bmj.com/site/about/guidelines.xhtml

growth and earlier onset of menarche are associated with greater CVD risk[20,21]. Shorter leg length and greater sitting height have also been associated with greater body mass index (BMI) and/or higher body fat percent[22,23]. It is therefore possible that the association between height components and CVD is influenced by body composition. Direct measurements of lean and fat mass reflect body composition better than BMI alone.

The Pune Children's Study (PCS) was set up to prospectively study early life antecedents of adult disease[24]. At 8 years of age, taller children had a more adverse CVD risk profile[24]. We have now investigated the relationship of final height and its components with CVD risk factors at 21 years of age (height components were not assessed at 8 years). We hypothesise: 1) Shorter leg length and greater sitting height will be associated with a more adverse CVD risk profile; 2) Body composition measurements (lean and fat mass) may explain the relationship between height components and CVD risk. To our knowledge, this has not been examined before. We also investigated CVD risk factors in relation to parental height and intergenerational change in height.

Methods

The PCS[17] is an urban cohort of 477 full-term singleton babies who were born in the KEM Hospital, Pune during 1987-1989. During 2009-11, the cohort members were invited for further studies. They were admitted to our unit the evening before the investigations and fasted overnight (~10 hours) after a standard dinner. Those who were pregnant at the time of invitation were seen ~6 months after delivery for investigations.

<u>Patient and public involvement</u>: It was not possible to involve participants in the design or conduct of the research project. The participants consisted of a birth cohort of men and women

For peer review only - http://bmjopen.bmj.com/site/about/guidelines.xhtml

born in the KEM Hospital and who had been followed up longitudinally since early childhood. Results of the investigations (where clinically relevant) have been reported back to participants and appropriate referrals arranged for those requiring clinical management. Summary results of the research findings have been shared with the cohort.

Anthropometry and body composition: Weight was measured to the nearest 5g using an electronic scale (Conweigh Electronic Instruments, Mumbai). Standing and sitting height were measured to the nearest 0.1 cm using a wall-mounted stadiometer. Leg length was computed by subtracting sitting height from standing height. The predicted height of the offspring (mid-parental height) was derived using a standard formula (boys: [{maternal height+paternal height}/2] + 7 cm; girls: [{maternal height+paternal height}/2] - 7 cm[25]. The intergenerational change in height between children and parents was calculated by subtracting mid-parental height from offspring adult height. Participants' total and regional body fat and lean mass were assessed using dual energy X-ray absorptiometry (DXA; Lunar Prodigy, GE, USA).

CVD risk factors: Blood pressure was measured using a digital monitor (UA 767PC, A & D Instruments Ltd, UK); the average of two readings made five minutes apart was used. Plasma lipids, glucose and insulin were measured on fasting venous blood. An oral glucose tolerance test was carried out according to the WHO protocol,[26] followed by further blood samples at 30 and 120 minutes for glucose and insulin.

The Standard of Living Index (SLI)[27] was used to assess socio-economic status. It is a standardized questionnaire based on information about housing, amenities and possessions; higher scores indicate higher social class. Ethical permission was obtained from the KEM Hospital Ethics Committee and informed consent was obtained from all participants.

For peer review only - http://bmjopen.bmj.com/site/about/guidelines.xhtml

Laboratory analyses: Plasma glucose, cholesterol, HDL-cholesterol, and triglyceride concentrations were measured using enzymatic methods (Hitachi 902, Germany). Between-batch coefficients of variation for all these assays were <3% in the normal range. Plasma insulin was measured using a Delfia technique (Victor 2, Wallac, Finland); between-batch coefficients of variation were <6%. Insulin resistance (HOMA-IR) and beta-cell function (HOMA-B) were calculated using the online Oxford model[28]. Insulin secretion was measured as Insulinogenic index (increment in plasma insulin/increment in plasma glucose at 30 min)(29]. The Matsuda index of insulin sensitivity was computed by k/sqrt(fasting glucose*120 min glucose*fasting insulin*120 min insulin) where k=10,000[30]. Disposition index was calculated as Insulinogenic index*Matsuda index, to reflect beta-cell function for concurrent insulin resistance as originally described by Bergman[31].

Definitions: Overweight was defined as BMI ≥25 kg/m^2 and <30 kg/m^2, and obesity as BMI ≥30 kg/m^2[32]. Stunting was defined as a height Z-score of less than -2 below the CDC average at 20 years of age[33]. Hyperglycaemia was defined as either impaired fasting glucose (IFG, fasting plasma glucose 5.6-6.9 mmol/L) or impaired glucose tolerance (IGT, 120-minute plasma glucose 7.8-11.0 mmol/L) or diabetes mellitus (DM, fasting plasma glucose ≥7.0 mmol/L or 120-minute plasma glucose ≥11.1 mmol/L)[34]. Hypercholesterolaemia was defined as plasma total cholesterol ≥5.1 mmol/L, [35] hypertriglyceridaemia as plasma triglyceride concentration ≥1.7 mmol/L and low HDL-cholesterol as HDL-cholesterol concentration <1.03 mmol/L for men and <1.29 mmol/L for women[36]. Hypertension was defined as systolic blood pressure ≥130 mmHg or diastolic blood pressure ≥85 mmHg[36].

For peer review only - http://bmjopen.bmj.com/site/about/guidelines.xhtml

Statistical Methods: Exposure variables (offspring height and its components, parental height, and intergenerational change in height) were converted into within-sample Z-scores to enable comparison of effects. Associations with body composition were tested using age-and-sex adjusted partial correlations. Multiple linear regression was used to identify associations between height components and CVD risk factors, adjusting for age, sex and age-sex interaction (model 1). We further adjusted for fat mass, lean mass and their interactions with sex (model 2). The estimated regression coefficients (β) represent the change in outcome per SD change in exposure. We examined the simultaneous association of height components and derived contour plots for the outcome variables as a function of leg length and sitting height, adjusted for age and sex. We used the Mahalanobis distance to identify the ellipse that contained 95% of the data, and to exclude unlikely combinations of leg length and sitting height.

To test possible selection bias, we compared body size, lipid and glucose-insulin variables between participants and non-participants using regression imputation. We developed an imputation model using multiple regression with variables significantly associated with each other at 8 years. We then applied this model to compare 21-year observed values for participants and imputed values for non-participants.

Statistical analyses were performed using SPSS 21 (SPSS Inc., USA) and R V.3.2.3.

Results

Of 477 children who were studied at 8 years of age, 357 (75%) participated in the 21-year study (191 males). Non-participants had higher 8-year BMI (14.0 v 13.6 kg/m^2, p=0.05), but similar 8-year height, and lipid, glucose and insulin concentrations compared to participants. Participants'

For peer review only - http://bmjopen.bmj.com/site/about/guidelines.xhtml

21-year BMI showed no differences from imputed values for non-participants (21.6 vs 21.9 kg/m^2; p>0.05).

General characteristics:

Both men and women had height CDC Z-scores below the average (Table 1). They were taller than their parents (men by 5.7 cm and women by 4.1 cm) with lower rates of stunting (8.0% and 14.5% of men and women versus 26.8% and 28.5% of fathers and mothers respectively). Compared to their fathers, sons had a greater leg length by 3.7 cm and sitting height by 2.5 cm. Compared to their mothers, daughters had a greater leg length by 2.5 cm and sitting height by 1.3 cm. Offspring height correlated positively with height of both parents (men: r=0.43 and 0.55 for fathers and mothers respectively; women: 0.50 and 0.48; p<0.001 for all). Heights of the parents were positively correlated (r=0.23; p<0.001). At 21 years, 18.5% of participants were overweight, 2.5% were obese, 4.8% were hypertensive, 5.6% had hypercholesterolaemia, 7.6% had hypertriglyceridaemia, and 69.3% had low HDL-cholesterol concentrations. None were on treatment for hypertension or dyslipidemia. Three participants known to have diabetes were on insulin therapy; a further 18.5% were found to be hyperglycaemic (11.2% IFG, 5.9% IGT and 1.4% type-2 DM).

Table 1: Characteristics of the cohort participants

General characteristics	Men (n=191)	Women (n=166)
**Birth data**		
Birth weight (g)	2824 (443)	2721 (481)
**21-year data**		
Age (years)	21.4 (0.4)	21.4 (0.4)
Anthropometry		
Height (cm)	172.0 (6.6)	156.8 (6.4)
CDC Z score	-0.66 (0.92)	-0.99 (0.98)

For peer review only - http://bmjopen.bmj.com/site/about/guidelines.xhtml

Stunted n (%)	15 (8.0)	24 (14.5)
Sitting height (cm)	89.8 (3.2)	82.5 (3.1)
Leg length (cm)	82.2 (4.4)	74.2 (4.3)
Leg length to sitting height ratio	0.91 (0.04)	0.89 (0.04)
Weight (kg)	65.3 (13.1)	52.3 (10.6)
BMI (kg/m^2)	22.0 (4.0)	21.2 (4.1)
Lean mass (kg)	46.5 (5.8)	30.2 (3.9)
Fat mass (kg)	15.7 (9.1)	19.1 (8.0)
Fat percent (%)	22.4 (9.6)	35.2 (8.4)
Cardiometabolic risk factors		
Systolic BP (mmHg)	115.3 (10.1)	102.9 (9.1)
Diastolic BP (mmHg)	65.4 (8.4)	63.6 (7.9)
Cholesterol (mmol/L)	3.8 (0.76)	3.75 (0.74)
Triglycerides (mmol/L)**	0.89 (0.67, 1.25)	0.76 (0.62, 1.03)
HDL cholesterol (mmol/L)	0.97 (0.17)	1.10 (0.22)
Fasting glucose (mmol/L)	5.1 (0.6)	5.0 (0.4)
120-minute glucose (mmol/L)**	5.5 (4.8, 6.5)	5.7 (4.8, 6.5)
Fasting insulin (pmol/L)**	41.4 (26.1, 64.2)	48.0 (31.8, 68.4)
HOMA-IR**	0.9 (0.6, 1.4)	1.0 (0.7, 1.5)
HOMA-B**	85.1 (60.2, 106.1)	94.5 (72.4, 122.5)
Insulinogenic Index**	52.2 (37.8, 78.2)	58.1 (39.1, 83.1)
Matsuda Index**	6.0 (3.8, 8.9)	5.2 (3.5, 8.0)
Disposition Index**	330.4 (191.5, 512.5)	290.9 (193.0, 520.3)
**Parental characteristics**		
	Fathers (n=310)	Mothers (n=360)
Age (years)	52.5 (4.2)	46.2 (4.5)
Height (cm)	165.9 (6.1)	153.0 (5.7)
Sitting height (cm)	87.3 (3.3)	81.4 (3.09)
Leg length (cm)	78.6 (4.0)	71.6 (3.7)
CDC Z score	-1.52 (0.84)	-1.58 (0.88)
Stunted n (%)	82 (26.8)	102 (28.5)

Values are mean (SD) unless otherwise stated; ** median (inter-quartile range)

Relationships of height with birth weight, socio-economic status, BMI and body composition:

Total height, leg length and sitting height at 21 years were positively associated with birthweight (r=0.39, 0.34 and 0.33 respectively; p<0.001 for all). Higher SLI scores were associated with

taller total height (r=0.11; p<0.05); correlations with leg length and sitting height were similar (0.09 and 0.10; p>0.05). Leg length and leg length to sitting height ratio were negatively associated with BMI (r=-0.18; and r=-0.30 respectively) while sitting height was positively associated (r=0.17); (p<0.01 for all). Total height and leg length were positively associated with lean mass while sitting height was positively associated with both lean and fat mass (Table 2). Leg length to sitting height ratio was inversely related to fat mass and body fat percent. We examined the relationship of height components with regional body composition measurements (arm lean and fat mass, leg lean and fat mass, and trunk lean and fat mass); the findings were similar to those seen with total lean and fat mass (Supplementary Figure 1). Height and leg length were positive associated with regional lean mass measurements while sitting height was positively associated with both regional lean and fat mass measurements. Leg length to sitting height ratio was negatively associated with regional fat mass measurements.

Table 2: Relationship of height and components of height to body composition

	Lean mass (kg)			Fat mass (kg)			Body fat percent		
	**β**	**95% CI**	**P**	**β**	**95% CI**	**p**	**B**	**95% CI**	**P**
**Men**									
Total height (z)	3.6	3.0, 4.3	<0.001	1.2	-0.1, 2.5	0.07	0.1	-1.3, 1.5	0.9
Leg length (z)	2.8	2.1, 3.6	<0.001	0.4	-1.0, 1.7	0.6	-0.5	-1.9, 0.9	0.5
Sitting height (z)	3.6	2.9, 4.3	<0.001	2.1	0.8, 3.4	0.001	1.0	-0.4, 2.4	0.1
Ratio of leg length to sitting height (z)	0.5	-0.3, 1.4	0.2	-1.2	-2.5, 0.1	0.08	-1.4	-2.7, 0.0	0.05
**Women**									
Total height (z)	2.0	1.5, 2.5	<0.001	1.1	-0.1, 2.3	0.08	-0.2	-1.5, 1.1	0.8
Leg length (z)	1.4	0.8, 1.9	<0.001	-0.6	-1.8, 0.7	0.4	-1.6	-2.9, -0.3	0.01
Sitting height (z)	2.3	1.8, 2.8	<0.001	2.8	1.6, 4.0	<0.001	1.5	0.2, 2.9	0.02
Ratio of leg length to sitting height (z)	0.0	-0.6, 0.6	0.9	-2.9	-4.0, -1.7	<0.001	-3.3	-4.5, -2.1	<0.001

Data derived using linear regression and all variables as continuous; All analyses were adjusted for age.

For peer review only - http://bmjopen.bmj.com/site/about/guidelines.xhtml

Relationships of height and its components with CVD risk factors:

Relationships between height components and CVD risk factors were similar in both sexes and there were no interactions with sex; we therefore present a sex-adjusted combined analysis. Total height was negatively associated with 120-minute glucose concentration and Matsuda index (Table 3). Leg length was negatively associated with diastolic blood pressure, total cholesterol, fasting and 120-minute glucose concentrations, and positively with Matsuda index. Sitting height was positively associated with systolic blood pressure and triglycerides. Leg length to sitting height ratio was negatively associated with systolic and diastolic blood pressure, total cholesterol, triglycerides, and fasting and 120-minute glucose concentrations, and positively associated with Matsuda index. After adjustment for lean and fat mass, the relationships of height and leg length with risk factors remained of similar strength except for Matsuda index, and a negative association of leg length with systolic blood pressure became apparent. The relationships of sitting height were significantly attenuated by body composition adjustment. The relationships of leg length to sitting height ratio were partially attenuated after adjustment for body composition. Regional body composition measurements (trunk fat and lean mass for sitting height, or leg fat and lean mass for leg length) did not attenuate these associations more than total fat and lean mass (data not shown). We then adjusted for all the regional body composition measurements instead of total lean and fat mass while examining the relationship between height components and cardiometabolic risk factors. There were no specific patterns with regional body composition measurements and these models did not explain the relationships between height components and CVD risk factors any more than total body composition measurements. These relationships remained similar after adjusting for birthweight and SLI.

For peer review only - http://bmjopen.bmj.com/site/about/guidelines.xhtml

Table 3: Relationship of components of height to CVD risk factors

		Exposure											
		Height (z)			Leg length (z)			Sitting height (z)			Leg length to sitting height (z)		
**Outcome**	**Model**	**B**	**95% CI**	**P**	**B**	**95% CI**	**P**	**B**	**95% CI**	**P**	**B**	**95% CI**	**P**
**Systolic BP (mmHg)**													
	1	0.36	(-0.66, 1.38)	0.49	-0.77	(-1.79, 0.25)	0.14	**1.79**	**(0.79, 2.80)**	**0.001**	**-2.18**	**(-3.18, -1.19)**	**<0.001**
	2	**-1.17**	**(-2.34, 0.00)**	**0.05**	**-1.74**	**(-2.82, -0.67)**	**<0.001**	0.33	(-0.87, 1.54)	0.58	**-1.74**	**(-2.73, -0.76)**	**<0.001**
**Diastolic BP (mmHg)**													
	1	-0.36	(-1.20, 0.49)	0.41	**-1.11**	**(-1.94, -0.27)**	**0.01**	0.77	(-0.07, 1.62)	0.07	**-1.82**	**(-2.65, -1.00)**	**<0.001**
	2	-0.50	(-1.46, 0.45)	0.30	**-0.93**	**(-1.81, -0.05)**	**0.04**	0.50	(-0.47, 1.47)	0.31	**-1.01**	**(-1.81, -0.20)**	**0.01**
**Total Cholesterol (mmol/l)**													
	1	-0.05	(-0.13, 0.04)	0.28	**-0.08**	**(-0.16, 0.00)**	**0.04**	0.02	(-0.06, 0.10)	0.64	**-0.11**	**(-0.19, -0.03)**	**0.009**
	2	-0.01	(-0.10, 0.09)	0.90	-0.05	(-0.13, 0.04)	0.30	0.05	(-0.05, 0.14)	0.33	-0.07	(-0.15, 0.01)	0.07
**Triglycerides (mmol/l)***													
	1	0.01	(-0.03, 0.06)	0.56	-0.02	(-0.06, 0.03)	0.48	**0.05**	**(0.00, 0.09)**	**0.04**	**-0.05**	**(-0.09, -0.01)**	**0.02**
	2	-0.01	(-0.06, 0.03)	0.59	-0.02	(-0.06, 0.02)	0.38	0.00	(-0.05, 0.05)	0.98	-0.02	(-0.06, 0.02)	0.37
**HDL-cholesterol (mmol/l)**													
	1	0.00	(-0.02, 0.02)	0.97	0.01	(-0.02, 0.03)	0.59	-0.01	(-0.03, 0.01)	0.52	0.01	(-0.01, 0.03)	0.23
	2	0.01	(-0.01, 0.04)	0.29	0.01	(-0.02, 0.03)	0.56	0.02	(-0.01, 0.05)	0.12	0.00	(-0.03, 0.02)	0.37
**Fasting glucose (mmol/l)**													
	1	-0.06	(-0.12, 0.01)	0.08	**-0.07**	**(-0.13, -0.01)**	**0.02**	-0.01	(-0.08, 0.05)	0.61	**-0.07**	**(-0.13, -0.01)**	**0.02**
	2	-0.04	(-0.12, 0.03)	0.28	-0.05	(-0.12, 0.02)	0.16	-0.01	(-0.09, 0.06)	0.71	-0.04	(-0.11, 0.02)	0.18
**120-min glucose (mmol/l)**													
	1	**-0.26**	**(-0.42, -0.10)**	**0.002**	**-0.31**	**(-0.47, -0.15)**	**<0.001**	-0.11	(-0.27, 0.06)	0.20	**-0.27**	**(-0.43, -0.11)**	**0.001**
	2	**-0.26**	**(-0.45, -0.07)**	**0.01**	**-0.21**	**(-0.39, -0.03)**	**0.02**	-0.17	(-0.37, 0.02)	0.08	-0.11	(-0.28, 0.05)	0.17
**Fasting insulin (pmol/l)***													
	1	0.00	(-0.08, 0.07)	0.92	-0.01	(-0.09, 0.06)	0.70	0.01	(-0.06, 0.08)	0.75	-0.03	(-0.10, 0.05)	0.47
	2	-0.01	(-0.09, 0.07)	0.82	0.02	(-0.06, 0.09)	0.70	-0.06	(-0.15, 0.02)	0.16	0.05	(-0.02, 0.12)	0.19
**HOMA-IR***													

For peer review only - http://bmjopen.bmj.com/site/about/guidelines.xhtml

	1	-0.01	(-0.08, 0.06)	0.79	-0.02	(-0.09, 0.05)	0.61	0.01	(-0.07, 0.08)	0.86	-0.03	(-0.10, 0.04)	0.44
	2	-0.01	(-0.09, 0.07)	0.82	0.02	(-0.06, 0.09)	0.69	-0.06	(-0.14, 0.03)	0.17	0.05	(-0.02, 0.12)	0.20
**HOMA-B**													
	1	-0.11	(-4.95, 4.73)	0.96	-0.93	(-5.76, 3.90)	0.71	1.04	(-3.80, 5.89)	0.67	-1.94	(-6.76, 2.89)	0.43
	2	-1.33	(-7.08, 4.43)	0.65	0.34	(-5.66, 4.98)	0.90	-3.69	(-9.57, 2.19)	0.22	1.78	(-3.10, 6.67)	0.47
**Insulinogenic index****													
	1	0.04	(-0.06, 0.15)	0.43	0.06	(-0.04, 0.17)	0.25	0.00	(-0.10, 0.11)	0.94	0.07	(-0.04, 0.17)	0.20
	2	0.08	(-0.05, 0.21)	0.24	0.10	(-0.02, 0.22)	0.12	0.00	(-0.14, 0.13)	0.95	0.09	(-0.02, 0.20)	0.11
**Matsuda index***													
	1	**0.08**	**(0.01, 0.16)**	**0.04**	**0.11**	**(0.03, 0.18)**	**0.005**	0.02	(-0.06, 0.09)	0.62	**0.11**	**(0.03, 0.18)**	**0.005**
	2	0.05	(-0.04, 0.13)	0.27	0.03	(-0.04, 0.11)	0.39	0.04	(-0.04, 0.13)	0.32	0.01	(-0.06, 0.08)	0.80
**Disposition index**													
	1	0.01	(-0.58, 0.60)	0.96	0.16	(-0.43, 0.74)	0.61	-0.18	(-0.77, 0.41)	0.55	0.32	(-0.27, 0.90)	0.29
	2	0.04	(-0.06, 0.14)	0.41	0.03	(-0.06, 0.12)	0.55	0.03	(-0.07, 0.12)	0.60	0.00	(-0.08, 0.08)	0.93

BP, blood pressure. * Outcome variables were log-transformed to ensure normality. ** Outcome variable was Fisher-Yates transformed to ensure normality Components of height were z-standardised. Model 1 was adjusted for age, sex and interaction of age and sex. Model 2 was adjusted for age, sex, interaction of age and sex, lean mass, fat mass, interaction of sex and lean mass, and interaction of sex and fat mass.

For peer review only

Relationships of CVD risk factors with leg length and sitting height simultaneously

The associations of leg length and sitting height simultaneously with CVD risk factors are shown in Table 3, and as contour plots in Figure 1 and Supplementary Figures 2 and 3. Because of the opposing relationships of leg length and sitting height with CVD risk factors, these associations tended to be accentuated. In general, at any leg length, greater sitting height was associated with a more adverse profile; at any sitting height, greater leg length was associated with a more favourable profile. The healthiest profiles tended to be in people with long legs and short sitting height. However, there was variation in patterns. For lean mass, systolic blood pressure, triglycerides, HDL-cholesterol, and fasting glucose (contour lines near-horizontal), the associations with sitting height were stronger than those with leg length (Figure 1). For 120-minute glucose and Matsuda index (contour lines near-vertical), the associations were stronger with leg length. For BMI, fat mass, total cholesterol and diastolic blood pressure (contour lines near-diagonal), the associations with leg length and sitting height, though in opposite directions, were of similar strength. There were no relationships with HOMA-IR, HOMA-B, Insulinogenic index or Disposition index.

Relationships with parental height and intergenerational change in height:

There were no relationships between height of either parent or mid-parental height with offspring CVD risk factors (Table 4). A greater increase in leg length between generations was associated with lower BMI, fasting insulin, and HOMA-IR while a greater increase in sitting height was associated with higher BMI. There were no interactions between parental and offspring heights on offspring CVD risk factors.

For peer review only - http://bmjopen.bmj.com/site/about/guidelines.xhtml

Table 4: Relationship of Parental height, and its components and intergenerational change in height with offspring CVD parameters at 21 years

Outcome at 21 years	Mid-parental height	Father's Height	Father's Leg length	Father's Sitting height	Mother's Height	Mother's Leg length	Mother's Sitting height	Intergenerational change in height	Intergenerational change in leg length	Intergenerational change in sitting height
BMI	0.07	0.03	-0.03	0.09	0.00	-0.04	0.05	-0.02	-0.13*	0.16**
SLI score	0.15**	0.13*	0.04	0.18**	0.10	0.07	0.10	0.00	0.05	-0.04
Systolic BP	0.07	0.04	-0.05	0.13*	0.06	0.01	0.10	-0.00	-0.09	0.03
Diastolic BP	0.02	0.02	-0.02	0.08	-0.04	-0.09	0.05	0.05	-0.09	0.03
Cholesterol	0.01	-0.03	-0.11	0.08	0.02	0.002	0.04	-0.06	-0.07	-0.01
Triglycerides	0.08	0.01	-0.01	0.04	0.04	-0.00	0.08	0.02	-0.03	0.06
HDL	-0.01	-0.02	-0.04	0.02	0.05	0.06	0.02	-0.01	0.02	-0.02
Fasting glucose	-0.04	-0.10	-0.08	-0.09	-0.00	-0.03	0.03	-0.06	-0.09	0.01
120-min glucose	-0.07	-0.10	-0.13*	-0.03	-0.05	-0.07	-0.01	-0.09	-0.09	-0.02
Fasting insulin	0.00	0.01	0.04	-0.03	0.01	-0.02	0.03	-0.09	-0.12*	-0.00
120-min insulin	0.02	-0.04	-0.05	-0.01	0.03	-0.04	0.09	-0.03	-0.05	0.02
HOMA-IR	0.00	0.00	0.04	-0.04	0.01	-0.02	0.04	-0.10	-0.13*	-0.00
HOMA-B	0.02	0.03	0.05	0.00	0.01	0.01	-0.01	-0.04	-0.06	0.04
Insulinogenic Index	0.11	0.11	0.13	0.03	0.05	0.05	0.03	-0.10	-0.03	-0.03
Matsuda Index	0.04	0.10	0.07	0.09	-0.01	0.01	-0.02	0.05	0.08	-0.05
Disposition Index	0.06	0.10	0.10	0.07	0.02	0.02	0.02	-0.07	0.00	-0.10

Values are Pearson correlation coefficients adjusted for age and gender, as appropriate; exposures and outcomes are Z-standardized.
*p<0.05, ** p<0.01,*** p<0.001

For peer review only - http://bmjopen.bmj.com/site/about/guidelines.xhtml

For peer review only

Discussion

There was an intergenerational increase in total height, leg length and sitting height in this young adult Indian urban cohort. Greater total adult height and leg length were significantly associated with lower BMI, greater lean body mass, and lower CVD risk factors, while a greater sitting height was significantly associated with higher BMI, lean and fat mass and higher CVD risk factors. These associations were accentuated when leg length and sitting height were examined simultaneously, and leg length to sitting height ratio was strongly negatively related to CVD risk factors. The relationships with sitting height were significantly attenuated by adjusting for lean and fat mass. Parental height was unrelated to offspring CVD risk factors; however a greater intergenerational increase in leg length was associated with lower insulin and insulin resistance.

The associations of shorter leg length with higher CVD risk factors in our study are consistent with findings from high-income countries which have shown inverse associations of leg length with blood pressure, glucose intolerance and dyslipidaemia[11-17]. The associations with sitting height are less consistent, with some studies showing positive associations with CVD risk factors (higher insulin resistance, lower HDL-cholesterol) and some showing no associations[11,12,14]. Shorter leg length has been associated with higher LDL-cholesterol, and greater sitting height with higher diabetes risk and dyslipidaemia in China[37] while a study in Ghanaian adults showed greater sitting height was associated with lower CVD risk[38]. Other studies in LMICs have shown no relationships between height components and CVD risk[5,6].

Similar to our findings, longer leg length has been associated with lower BMI, and greater sitting height with higher BMI[22,23]. No previous studies have examined associations between components of adult height and CVD risk in relation to direct measurements of fat and lean mass. While longer leg length was associated with greater lean mass and lower body fat

For peer review only - http://bmjopen.bmj.com/site/about/guidelines.xhtml

percent, this did not appear to explain the inverse association between leg length and CVD risk factors – only the relationship between leg length and Matsuda index was attenuated after adjustment for lean and fat mass. In contrast, our data indicate that the relationship between sitting height and CVD risk factors could be partly explained by higher fat mass.

Secular increases in height in high-income countries are now slowing down[39]. Much of this increase is reflected in greater leg length[7]; this increment has been shown to occur by 2 years of age[7,40,41]. Leg length increases faster than sitting height in infancy suggesting that it may be related to better fetal and/or infant health and nutrition[42]. In contrast, sitting height increases more than leg length in later childhood,[40-42] due to faster growth of vertebrae than long bones during the pubertal growth spurt[19,22,40,41]. However, direct evidence that leg length and sitting height reflect conditions in early life and later childhood respectively is lacking. Birth weight, which might be expected to predict longer leg length, is equally strongly predictive of sitting height,[12,15] as in our study. The independent relationships of leg length and sitting height with CVD risk factors suggest that patterns and determinants of linear growth are important and may also influence body composition and CVD risk.

For peer review only

Our data do not elucidate the mechanisms linking greater sitting height to increased adiposity or greater leg length to lower CVD risk. Lower birth and infant weight are associated with lower adult lean mass, while greater weight gain in later childhood is associated with increased adult adiposity in several countries including LMICs[43]. Both are associated with a higher prevalence of CVD, type 2 diabetes and their risk factors[44,45]. These findings led to the developmental programming hypothesis that early undernutrition permanently alters the body's structure and metabolism leaving an increased vulnerability to the adverse effects of 'excess' nutrition in later life[46]. This sequence has been suggested as a possible explanation for the rising epidemics of CVD and diabetes, and high risk factor levels (as exemplified by our cohort), in LMICs. If it is

For peer review only - http://bmjopen.bmj.com/site/about/guidelines.xhtml

true that lower leg length reflects poorer early life environment, and greater sitting height reflects accelerated puberty and increased adiposity, our findings linking CVD risk factors to these height components would be broadly consistent with the developmental programming hypothesis. Our findings do not suggest that the relationship between short height and CVD risk reflects lower socio-economic status. Firstly, we found no relationships of socio-economic status with CVD risk factors. Secondly, we would expect both short leg length and short sitting height to be associated with higher CVD risk. Although genetic explanations for the associations between components of height and CVD risk factors are possible, common genes that influence both height and CVD explain little of the effect[47]. We have not explored genetic determinants in our study. Given the age of our cohort, reverse causality is unlikely to be a factor.

A greater increase in leg length between generations was associated with lower fasting insulin and insulin resistance. No previous studies have examined changes in leg length or sitting height between generations. A study in Scotland showed that although short height of both parents was independently associated with increased risk of coronary heart disease in the offspring, the association was stronger with mothers' height[48]. A study from India showed that shorter maternal but not paternal height was associated with higher risk of glucose intolerance in the offspring[49]. A greater increase in leg length between generations may reflect better fetal and infant growth and this may have persisting effects on offspring development and health. This may also be reflected through greater insulin sensitivity promoting greater growth.

The PCS is one of few cohorts in a LMIC with follow-up from childhood to adult life and measurements of adult height components and parental heights. Our loss to follow up of ~25% is low for a long term longitudinal study and adds to internal validity. Our attrition was mainly due to loss to follow-up in urban Pune with very few refusals. Moreover our study sample was similar in key characteristics to those who were lost to follow up. Limitations were a lack of data

For peer review only - http://bmjopen.bmj.com/site/about/guidelines.xhtml

on length, and on height components at birth and longitudinally throughout the growing period. While we have direct measurements of lean and fat mass, we are unable to differentiate between visceral and parietal fat components. The participants were all born in one hospital in Pune and who were available for follow-up from childhood to young adulthood; this limits representativeness given the population size and socio-economic diversity of India. However the KEM is the second largest hospital in Pune and offers services to a wide range of socio-economic classes.

Conclusions*:* Ours is the first study to examine associations of CVD risk factors with components of adult height, using body composition measures to understand the relationship. Shorter leg length and greater sitting height were associated with a more adverse CVD risk factor profile. We speculate that the associations with leg length and sitting height reflect the benefits of better growth in early childhood which is protective, and accelerated growth in later childhood which may be detrimental, and that these relationships are partially explained by body composition. Perhaps the strongest message that emerges from this analysis is the need to gain a better understanding of the determinants of height components.

Acknowledgments: We are grateful to the study participants for taking part in this study. We thank Dr KJ Coyaji, Medical Director of the KEM Hospital and Dr VS Padbidri, Director Research for providing research facilities. We thank Mrs PC Yajnik, Ms LV Ramdas, Ms Sonali Hardikar, Ms Sonali Wagle, Ms.Vaishali Deshpande, Ms Nilam Memane, Ms Deepa Raut, Ms Pallavi Hardikar, Ms Sonal Kasture, Ms Vaishali Kantikar, Ms Komal Advani, Ms Neha Gurav, Ms Rupali Saswade and Mr Arjunsing Patilkendre for their contribution in collection of the data. We also thank Mr TM Deokar, Mr SD Chougule, Mr AB Gaikwad, Mr ML Hoge, Mr. Vipul Wagh, Mr SN Khemkar, Mr SB Wagh, Mr BS Jadhav and Ms Prachi Katre for their invaluable

For peer review only - http://bmjopen.bmj.com/site/about/guidelines.xhtml

contribution to the study. Dr Sharad Gore helped with statistical queries. We also acknowledge the support of Sneha-India.

Funding: The Study is funded by The Wellcome Trust, UK (Grant Number: 083460/Z/07/Z), the Medical Research Council, UK and the Department for International Development, UK.

Conflict of interest: None of the authors had any financial or personal conflicts of interest associated with this manuscript.

Contributor statement: CSY and CHDF designed the study, analysed and interpreted the data and wrote the manuscript. KK, SMJ and CDG analysed and interpreted the data, and wrote the manuscript. HL, DSB, CJ and ASK carried out data collection and critically reviewed the manuscript. CO analysed and interpreted the data, and critically reviewed the manuscript. AB, SAB and ANP designed the study and critically reviewed the manuscript. All authors approved the final version. CSY is the guarantor of this work and as such, had full access to all the data in the study and takes responsibility of the integrity of the data and the accuracy of the data analysis.

Data availability statement: Data are available upon reasonable request. The cohort is a hospital-based birth cohort with data collected at 4, 8 and 21 years on anthropometry, dietary information and cardiometabolic risk factors. Data collected earlier at birth, 4 and 8 years have been published and we are in the process of analysing and publishing data collected at 21 years. For access to data, please contact Prof CS Yajnik, Director, Diabetes Unit, KEM Hospital Research Centre, Pune, India. E-mail: diabetes@kemdiabetes.org; csyajnik@gmail.com.

For peer review only - http://bmjopen.bmj.com/site/about/guidelines.xhtml

References:

1. The Emerging Risk Factors Collaboration. Adult height and the risk of cause-specific death and vascular morbidity in 1 million people: individual participant meta-analysis. *Int J Epidemiol* 2012;**41**:1419-33.
2. Davey Smith G, Hart C, Upton M et al. Height and risk of men among men and women: aetiological implications of associations with cardiovascular disease and cancer mortality. *J Epidemiol Community Health* 2000;**54**:97-103.
3. Paajanen TA, Oksala NKJ, Kuukasjarvi P et al. Short stature is associated with coronary heart disease: a systematic review of the lierature and a meta-analysis. *Eur Heart J* 2010;**31**:1802-1809.
4. Man Ying Lee C, Barzi F, Woodward M et al. Adult height and the risks of cardiovascular disease and major causes of death in the Asia-Pacific region: 21000 deaths in 510000 men and women. *Int J Epidemiol* 2009;**38**:1060-1071.
5. Song YM, Davey Smith G, Sung J. Adult height and cause- specific mortality: a large cohort study of South Korean men. *Am J Epidemiol* 2003;**158**:479-485.
6. Sichieri R, Siqueira KS, Pereira RA et al. Short stature and hypertension in the city of Rio de Janeriro, Brazil. *Public Health Nutr* 2003;**3**:77-82.
7. Cole TJ. Secular trends in growth. *Proc Nutr Soc* 2000;**59**:317-24.
8. Sorensen HT, Sabroe S, Rothman KJ et al. Birth weight and length as predictors for adult height. *Am J Epidemiol* 1999;**149**:726-729.
9. Barker DJP, Osmond C, Winter PD et al. Weight in infancy and death from ischaemic heart disease. *Lancet* 1989;**ii**:577-80.
10. Marmot MG, Shipley MJ, Rose G. Inequalities in death – specific explanations of a general pattern? *Lancet* 1984;**i**:1003-6.
11. Batty GD, Shipley MJ, Gunnell D, et al. Height loss and future coronary heart disease in London: the Whitehall II study. *J Epidemiol Community Health* 2011;**65**:461-4.

For peer review only - http://bmjopen.bmj.com/site/about/guidelines.xhtml

12. Davey Smith G, Greenwood R, Gunnell D et al. Leg length, insulin resistance and coronary heart disease: The Caerphilly Study. *J Epidemiol Community Health* 2001;**55**:867-872.

13. Lawlor DA, Taylor M, Davey Smith G et al. Associations of components of adult height with coronary heart disease in postmenopausal women: The British Women's Heart and Health Study. *Heart* 2004;**90**:745-749.

14. Whitley E, Martin RM, Davey Smith G et al. The association of childhood height, leg length and other measures of skeletal growth with adult cardiovascular disease: the Boyd-Orr cohort. *J Epidemiol Community Health* 2012;**66**:18-23.

15. Lawlor DA, Ebrahim S, Davey Smith G. The association between components of adult height and type II diabetes and insulin resistance: the British Women's Heart and Health Study. *Diabetologia* 2002;**45**:1097-1106.

16. Wittenbecher C, Kluxhaus O, Boeing H et al. Associations of short stature and components of height with incidence of type 2 diabetes: mediating effects of cardiometabolic risk factors. *Diabetologia* 2019;**62**:2211–21.

17. Asao K, Kao WH, Baptiste-Roberts K, et al. Short stature and the risk of adiposity, insulin resistance, and type 2 diabetes in middle age: the Third National Health and Nutrition Examination Survey (NHANES III), 1988-1994. *Diabetes Care* **2006**;29:1632-7.

18. Barker DJP. Fetal origins of coronary heart disease. *BMJ* 1995;**311**:171-4.

19. Frisancho AR. Relative Leg Length as a Biological Marker to Trace the Developmental History of Individuals and Populations: Growth Delay and Increased Body Fat. *Am J Hum Biol* 2007;**19**:703–10.

20. Singhal A, Lucas A. Early origins of cardiovascular disease: is there a unifying hypothesis? *Lancet* 2004;**263**:1642-5.

21. Horta BL, Barros FC, Victora CG, et al. Early and late growth and blood pressure in adolescence. *J Epidemiol Community Health* 2003;**57**:226-30.

For peer review only - http://bmjopen.bmj.com/site/about/guidelines.xhtml

22. Lorentzon M, Norjavaara E, Kindblom JM. Pubertal timing predicts leg length and childhood body mass index predicts sitting height in young adult men. *J Pediatr* 2011;**158**:452-7.

23. Velasquez-Melendez G, Silveira EA, Allencastro-Souza P et al. Relationship between sitting-height-to-stature ratio and adiposity in Brazilian women. *Ann Hum Biol* 2005;**17**:646-53.

24. Bavdekar A, Yajnik CS, Fall CH et al. Insulin resistance syndrome in 8-year-old Indian children: small at birth, big at 8 years, or both? *Diabetes* 1999;**48**:2422-2429.

25. Freeman JV, Cole TJ, Chinn S et al. Cross-sectional stature and weight reference curves for the UK, 1990. *Arch Dis Child* 1995;**73**:17-24.

26. Definition, Diagnosis and Classification of Diabetes Mellitus and its Complications Report of a WHO Consultation. Part 1: Diagnosis and Classification of Diabetes Mellitus. Geneva: World Health Organization; 1999.

27. International Institute for Population Sciences and Macro International. National Family Health Survey (NHFS-3), India 2005-06. Mumbai: IIPS; 2007.

28. The Oxford Centre for Diabetes, Endocrinology and Metabolism, Diabetes Trials Unit. HOMA calculator. Available from http://www.dtu.ox.ac.uk, accessed 1 July 2014.

29. Wareham NJ, Phillips DI, Byrne CD et al. The 30 minute insulin incremental response in an oral glucose tolerance test as a measure of insulin secretion. *Diabet Med* 1995;**12**:931.

30. DeFronzo RA, Matsuda M. Reduced time points to calculate the composite index. *Diabetes Care* 2010;**33**:e93

31. Bergman RN, Phillips LS, Cobelli C. Physiologic evaluation of factors controlling glucose tolerance in man. Measurement of insulin sensitivity and β-cell glucose sensitivity from the response to intravenous glucose. *J Clin Invest* 1981;68:1456–67.

For peer review only

For peer review only - http://bmjopen.bmj.com/site/about/guidelines.xhtml

32. BMI classification. World Health Organization. Global database on body mass index.Available from http://www.who.int/bmi, accessed 15 July 2014.

33. Centre for Disease Control and Prevention. www.cdc.gov/growthcharts/zscore.htm, accessed 15 July 2014.

34. American Diabetes Association. Standards of medical care in diabetes – 2013. *Diabetes Care* 2013;**36**:S11-66.

35. National Cholesterol Education Program Expert Panel on Detection, Evaluationand Treatment of High Blood Cholesterol in Adults (Adult Treatment Panel III). 2002. (Publication No. NIH 02:5215).

36. Alberti KG, Zimmet P, Shaw J. Metabolic syndrome - a new world-wide definition. A Consensus Statement from the International Diabetes Federation. *Diabet Med.* 2006;**23**:469-480.

37. Schooling CM, Jiang C, Lam TH et al. Height, its components and cardiovascular risk among older Chinese: a cross-sectional analysis of the Guangzhou Biobank Cohort Study. *Am J Public Health* 2007;**97**:1834-1841.

38. Boateng D, Danquah I, Said-Mohamed R et al. Early-life exposures and cardiovascular disease risk among Ghanaian migrant and home populations: The RODAM study. *J DOHAD* 2020;**11**:250-63.

39. Larnkjaer A, Schroder SA, Schmidt IM et al. Secular change in adult stature has come to a halt in northern Europe and Italy. *Acta Paediatr* 2006;**96**:754-755.

40. Tanner JM, Hayashi T, Preece MA et al. Increase in growth of leg relative to trunk in Japanese children and adults from 1957 to 1977: comparison with British and with Japanese Americans. *Ann Hum Biol* 1982;**9**:411-23.

41. Cameron N, Tanner JM, Whitehouse RH. A longitudinal analysis of the growth of limb segments in adolescence. *Ann Hum Biol* 1982;**9**:211-20.

For peer review only

For peer review only - http://bmjopen.bmj.com/site/about/guidelines.xhtml

42. Gunnell D. Commentary: Early insights into height, leg length, proportionate growth and health. *Int J Epidemiol* 2001;**30**:221-2.

43. Kuzawa CW, Hallal PC, Adair L at al. Birth weight, postnatal weight gain, and adult body composition in five low and middle income countries. *Am J Hum Biol* 2012;**24**:5-13.

44. Eriksson JG, Forsen T, Tuomilehto HJ et al. Early growth and coronary heart disease in later life: longitudinal study. *BMJ* 2001;**322**:949-53.

45. Bhargava SK, Sachdev HPS, Fall CHD et al. Relation of serial changes in childhood body mass index to impaired glucose tolerance in young adulthood. *New England J Med* 2004;**350**:865-75.

46. Barker DJP, Gluckman PD, Godfrey KM et al. Fetal nutrition and cardiovascular disease in adult life. *Lancet*1993;**341**:938-41.

47. Nelson CP, Hamby SE, Saleheen D, et al. Genetically determined height and coronary artery disease. *New Engl J Med* 2015;**372**:1608-18.

48. Gray L, Davey Smith G, McConnachie A et al. Parental height in relation to offspring coronary heart disease: examining transgenerational influences on health using the west of Scotland Midspan Family Study. *Int J Epidemiol* 2012;**41**:1776-1785.

49. Raghupathy R, Antonisamy B, Geethanjali FS et al. Glucose tolerance, insulin resistance and insulin secretio in young south Indian adults: relationship to parental size, neonatal size and childhood body mass index. *Diab Res Clin Pract* 2010;**87**:283-92.

For peer review only

For peer review only - http://bmjopen.bmj.com/site/about/guidelines.xhtml

Figure 1: Relationships of leg length and sitting height with selected CVD risk factors

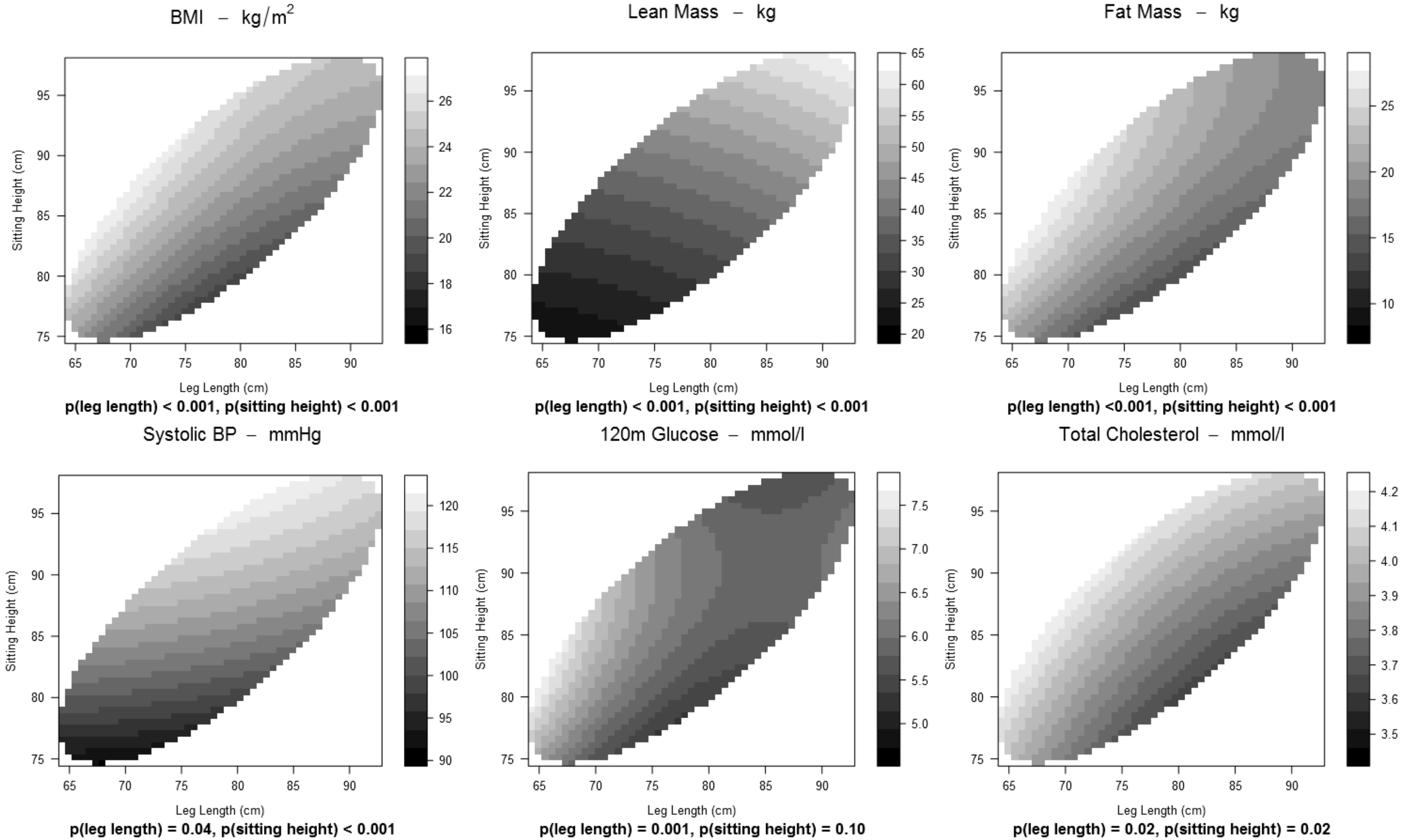

Supplementary Figure 1: Matrix showing correlations between height components and regional body composition measures

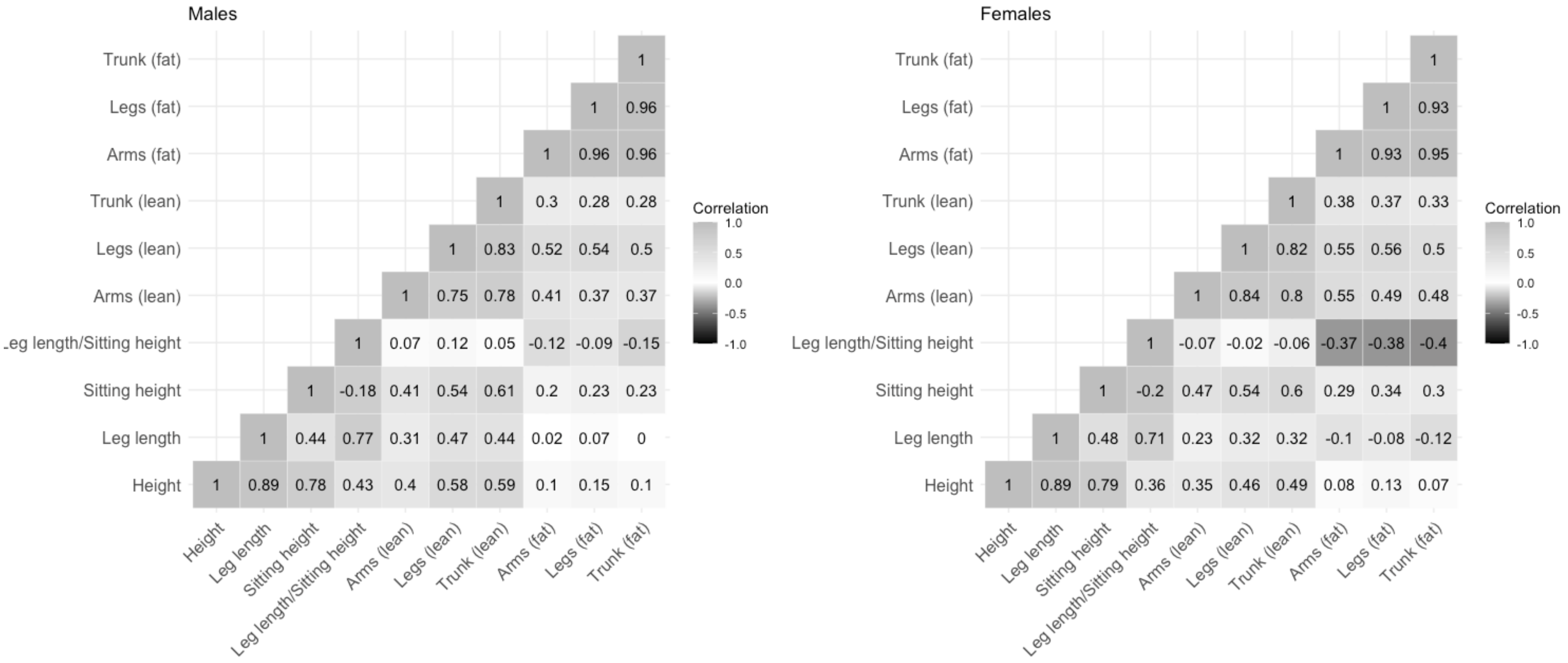

Supplementary Figure 2: Relationships of leg length and sitting height with selected CVD risk factors

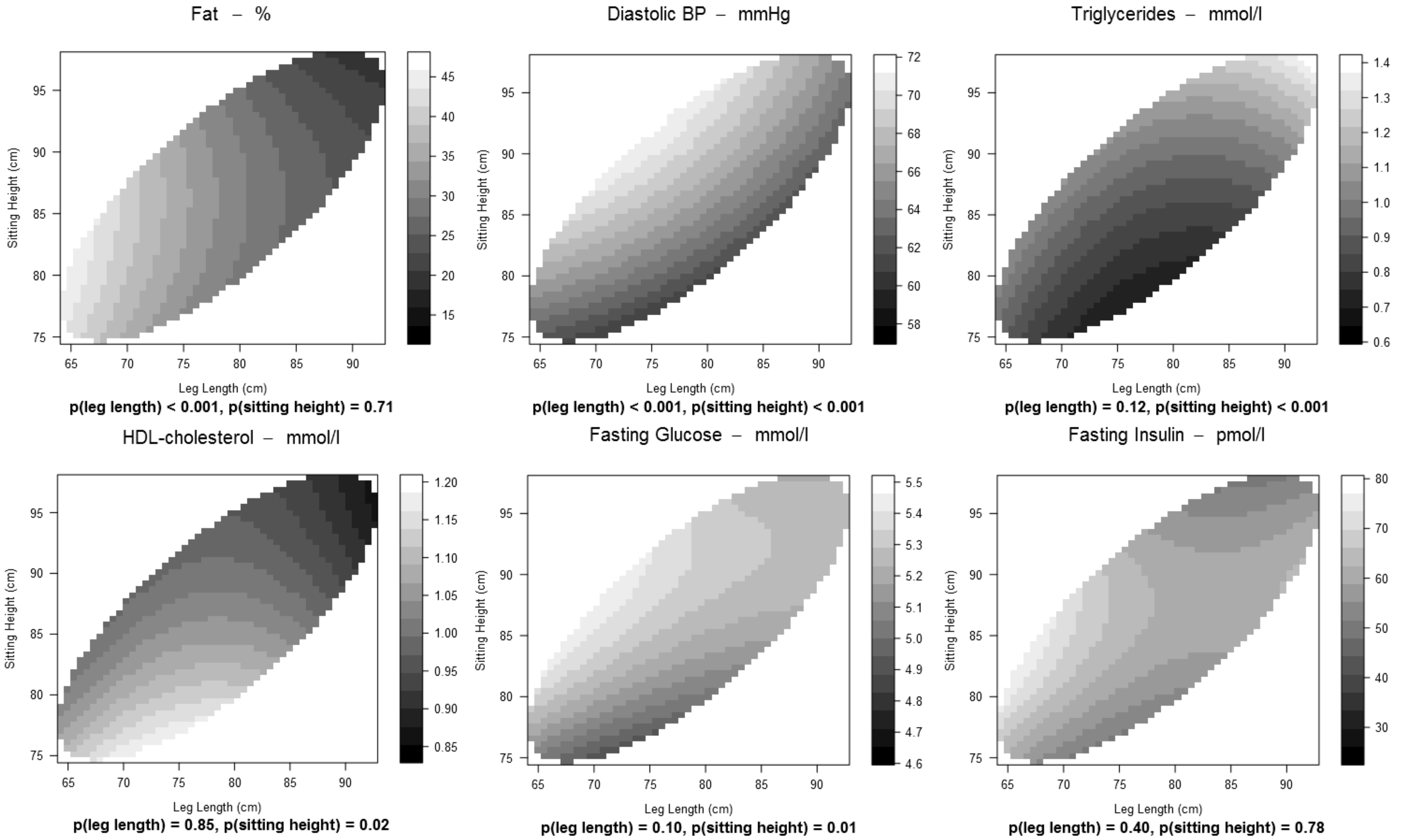

Supplementary Figure 3: Relationships of leg length and sitting height with selected CVD risk factors

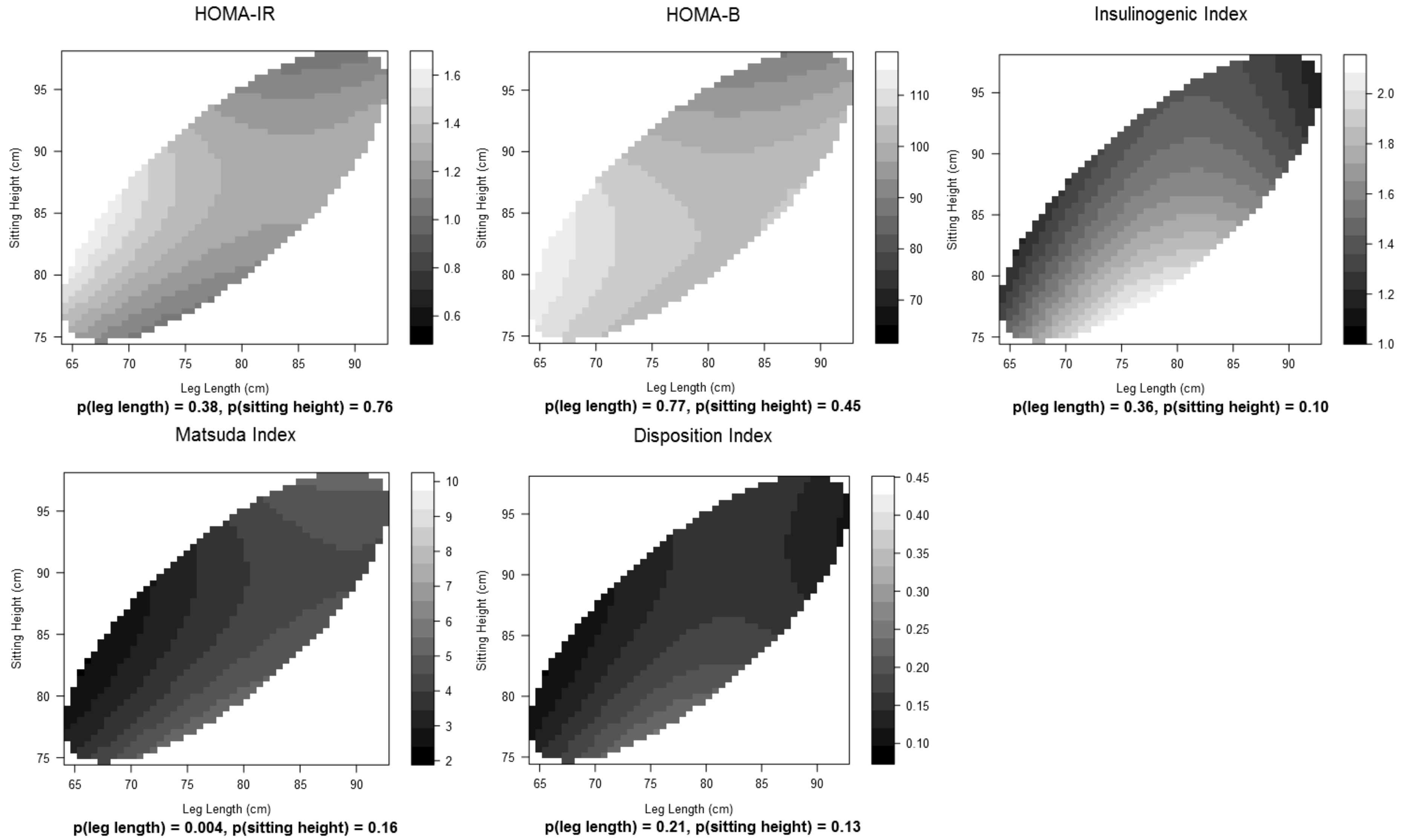

STROBE Statement—checklist of items that should be included in reports of observational studies

	Item No	Recommendation	Page number
**Title and abstract**	1	(*a*) Indicate the study's design with a commonly used term in the title or the abstract	1-2
		(*b*) Provide in the abstract an informative and balanced summary of what was done and what was found	2
**Introduction**			
Background/rationale	2	Explain the scientific background and rationale for the investigation being reported	4-5
Objectives	3	State specific objectives, including any prespecified hypotheses	5
**Methods**			
Study design	4	Present key elements of study design early in the paper	5
Setting	5	Describe the setting, locations, and relevant dates, including periods of recruitment, exposure, follow-up, and data collection	5-6
Participants	6	(*a*) *Cohort study*—Give the eligibility criteria, and the sources and methods of selection of participants. Describe methods of follow-up<br>*Case-control study*—Give the eligibility criteria, and the sources and methods of case ascertainment and control selection. Give the rationale for the choice of cases and controls<br>*Cross-sectional study*—Give the eligibility criteria, and the sources and methods of selection of participants	5
		(*b*) *Cohort study*—For matched studies, give matching criteria and number of exposed and unexposed<br>*Case-control study*—For matched studies, give matching criteria and the number of controls per case	
Variables	7	Clearly define all outcomes, exposures, predictors, potential confounders, and effect modifiers. Give diagnostic criteria, if applicable	5-7
Data sources/ measurement	8*	For each variable of interest, give sources of data and details of methods of assessment (measurement). Describe comparability of assessment methods if there is more than one group	5-7
Bias	9	Describe any efforts to address potential sources of bias	8
Study size	10	Explain how the study size was arrived at	5
Quantitative variables	11	Explain how quantitative variables were handled in the analyses. If applicable, describe which groupings were chosen and why	7-8
Statistical methods	12	(*a*) Describe all statistical methods, including those used to control for confounding	7-8
		(*b*) Describe any methods used to examine subgroups and interactions	7-8
		(*c*) Explain how missing data were addressed	8
		(*d*) *Cohort study*—If applicable, explain how loss to follow-up was addressed<br>*Case-control study*—If applicable, explain how matching of cases and controls was addressed<br>*Cross-sectional study*—If applicable, describe analytical methods taking account of sampling strategy	8
		(*e*) Describe any sensitivity analyses	8

Continued on next page

For peer review only - http://bmjopen.bmj.com/site/about/guidelines.xhtml

**Results**			
Participants	13*	(a) Report numbers of individuals at each stage of study—eg numbers potentially eligible, examined for eligibility, confirmed eligible, included in the study, completing follow-up, and analysed	8
		(b) Give reasons for non-participation at each stage	8
		(c) Consider use of a flow diagram	
Descriptive data	14*	(a) Give characteristics of study participants (eg demographic, clinical, social) and information on exposures and potential confounders	8-9
		(b) Indicate number of participants with missing data for each variable of interest	8-9
		(c) *Cohort study*—Summarise follow-up time (eg, average and total amount)	
Outcome data	15*	*Cohort study*—Report numbers of outcome events or summary measures over time	8-9
		Case-control study—Report numbers in each exposure category, or summary measures of exposure	
		Cross-sectional study—Report numbers of outcome events or summary measures	
Main results	16	(*a*) Give unadjusted estimates and, if applicable, confounder-adjusted estimates and their precision (eg, 95% confidence interval). Make clear which confounders were adjusted for and why they were included	8-16
		(*b*) Report category boundaries when continuous variables were categorized	
		(*c*) If relevant, consider translating estimates of relative risk into absolute risk for a meaningful time period	
Other analyses	17	Report other analyses done—eg analyses of subgroups and interactions, and sensitivity analyses	8-16
**Discussion**			
Key results	18	Summarise key results with reference to study objectives	17
Limitations	19	Discuss limitations of the study, taking into account sources of potential bias or imprecision. Discuss both direction and magnitude of any potential bias	19-20
Interpretation	20	Give a cautious overall interpretation of results considering objectives, limitations, multiplicity of analyses, results from similar studies, and other relevant evidence	20
Generalisability	21	Discuss the generalisability (external validity) of the study results	19-20
**Other information**			
Funding	22	Give the source of funding and the role of the funders for the present study and, if applicable, for the original study on which the present article is based	21

*Give information separately for cases and controls in case-control studies and, if applicable, for exposed and unexposed groups in cohort and cross-sectional studies.

Note: An Explanation and Elaboration article discusses each checklist item and gives methodological background and published examples of transparent reporting. The STROBE checklist is best used in conjunction with this article (freely available on the Web sites of PLoS Medicine at http://www.plosmedicine.org/, Annals of Internal Medicine at http://www.annals.org/, and Epidemiology at http://www.epidem.com/). Information on the STROBE Initiative is available at www.strobe-statement.org.

For peer review only - http://bmjopen.bmj.com/site/about/guidelines.xhtml
